# From Binding to Building: A Squaramide-Based Ion Pair Receptor as an Iniferter for Functional Polymer Synthesis

**DOI:** 10.3390/molecules30163362

**Published:** 2025-08-13

**Authors:** Mikołaj Prokopski, Marta Zaleskaya-Hernik, Wojciech Witkowski, Piotr Garbacz, Jan Romański

**Affiliations:** Faculty of Chemistry, University of Warsaw, Pasteura 1, PL 02-093 Warsaw, Poland; m.prokopski@student.uw.edu.pl (M.P.); mzaleskaya@chem.uw.edu.pl (M.Z.-H.); w.witkowski3@uw.edu.pl (W.W.); pgarbacz@uw.edu.pl (P.G.)

**Keywords:** ion pair receptors, host-guest interactions, inifreter, squaramide, functional polymers

## Abstract

To address the challenge of developing the first squaramide-based ion pair receptor acting as an iniferter in the polymerization process, a well-known BDPA molecule with specific radical functions was incorporated into its structure. The developed ditopic receptor demonstrated the ability to cooperatively bind ion pairs. Moreover, it proved to be an effective iniferter in the polymerization reaction using methyl methacrylate. The polymerization process preserved the ion-binding properties of the receptor, enabling the formation of functional polymeric materials. It was shown that the resulting polymer with the embedded receptor can be used for salt extraction from both solid and liquid phases, whereas the reference receptor lacking the BDPA unit did not exhibit this capability. This opens new avenues for the design of intelligent and selective polymeric materials for applications in supramolecular chemistry and separation technologies.

## 1. Introduction

The 1,3-bis(diphenylene)-2-phenylallyl (BDPA) radical, also known as the Koelsch radical, is an air-stable, carbon centered radical, synthesized for the first time by C. F. Koelsch in the 1930s, although the paper describing its synthesis was not published at that time, as the reviewer stated that described properties could not be those of a radical [1]. Only 25 years later, quantum mechanical computations proved that a radical of such a structure is indeed exceptionally stable and that properties of the BDPA radical described in the first paper would not be inconsistent with its structure [2]. R. Kuhn and A. Neugebauer improved the synthesis in 1964 [3], eliminating the usage of toxic mercury, greatly improving the yield of the final radical, and setting the standard for all modern synthesis of BDPA analogs. The BDPA radical finds its main uses as a molecular standard in EPR and electron nuclear double resonance (ENDOR) and as a polarizing agent in dynamic nuclear polarization (DNP) NMR experiments [4,5,6,7]. Unfortunately, because of its apolar character, BDPA radicals are poorly soluble in water, which renders them inferior to other stable radicals used in DNP NMR, such as trityl radicals of which some water-soluble analogs are known. To combat this issue, T. M. Swager et al. synthesized sodium salts of sulfonated and carboxylated BDPA derivatives of greatly improved water solubility while retaining the characteristics required for polarization in DNP NMR [8,9]. There were also attempts to improve the spectral characteristics necessary for DNP NMR, which resulted in the synthesis of a series of BDPA radical derivatives incorporating additional radical moieties in its structures [10,11]. Finally, S. Sigurdsson et al. combined these two ideas and synthesized BDPA-TEMPO biradical, which included tetramethylammonium functionalized fluorene groups. This approach guaranteed both high polarization efficiency and water solubility and additionally improved persistence to conclude research in this direction so far [12]. Apart from that, electrical characterization studies were carried out to determine the potential of the BDPA radical as an organic semiconductor [13], which was followed by the synthesis of a semiconducting radical polymer displaying ambipolar redox activity and the Faraday effect [14]. Finally, E. Lattmann et al. synthesized a 4,5-diazafluorene derivative of the Koelsch radical capable of forming a stable complex with CuCl_2_, representing the first reported metal complex of a free radical [15].

**Figure 1:**
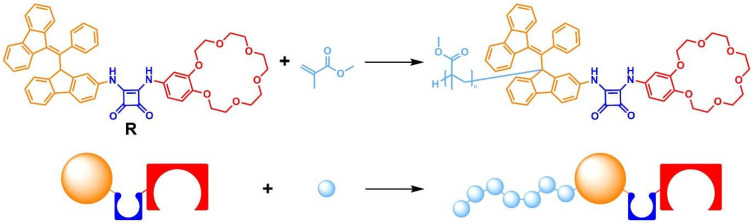
Schematic representation of methyl methacrylate polymerization. Light-blue denotes the polymer chain; orange, the iniferter; dark blue, the anion-binding domain; red, the cation-binding domain.

The BDPA radical and the aforementioned trityl radicals share a number of properties, including spectroscopic characteristics, the atom on which the radical is centered, and the way their structure stabilizes them. Iniferters are a group of compounds that are able to simultaneously act as initiators, transfer agents, and terminators in radical polymerization reactions and provide an efficient way of ensuring a linear relationship of polymer parameters with reaction time without the need for additional transition metal salt additives, as in the case of ATRP [16,17,18]. In 2008, Y. Xu et al. demonstrated that triphenylmethanes, due to their low bond dissociation energy and amphihydric character, can be used as iniferters in radical polymerization of methyl methacrylate [19,20,21]. Given the similarities of trityl and BDPPA radicals, we assumed that BDPA should be able to efficiently fill the role of iniferter in this reaction. Since radical polymerization should not disturb the structure of BDPA other than substituting one of its central hydrogen atoms with a polymer chain, it suggests that this approach opens up the possibility of fabricating monofunctionalized polymers. In most systems presented in the literature where a salt receptor is incorporated into a copolymer, the receptor has a pendant character or an incorporation group into the rigid architecture of the polymer [22,23,24,25]. On the other hand, within the family of ion pair receptors, those employing squaramide units for anion binding have attracted significant attention [26]. The choice of squaramide as the anion-binding motif is dictated by its superior binding affinity compared to urea or thiourea analogues, as well as by its compatibility with modular synthesis strategies, which streamline the preparation of functional materials. However, systems relying on hydrogen bond donors and acceptors, such as squaramides, often suffer from poor solubility. To address this issue, the incorporation of ion pair receptor units into polymeric frameworks has been shown to overcome the solubility limitations of free squaramides, thereby enabling their application in salt extraction processes [27]. Nevertheless, to the best of our knowledge, no ion pair receptor based on a squaramide acting as an iniferter in a polymerization reaction has been reported so far. To fill this methodological gap, herein we report the synthesis of a squaramide-based ion pair receptor equipped with a BDPA unit, investigate its binding abilities towards anions and ion pairs, and apply this compound as an iniferter in the polymerization reaction using methyl methacrylate (Figure 1).

## 2. Results and Discussion

### 2.1. Design and Synthesis

**Scheme 1:**
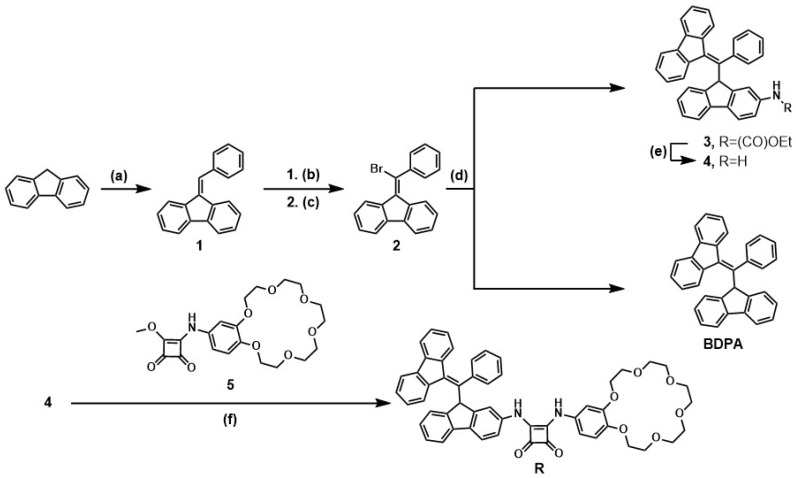
Synthesis scheme of receptor **R** and **BDPA**. Reagents and conditions: (a) *t*-BuOK 5 eq, EtOH(dry), Ar, 80°C, 83 %; (b) HBr/AcOH (38%), r.t., 84%; (c) NaOH 4 eq, EtOH(dry), Ar, 80°C, 84%; (d) fluorene 1 eq for **BDPA** (42%), ethyl N-(fluorenyl)carbamate 1 eq for 3 (50%), *t*-BuOK 5 eq, THF(dry), Ar, r.t.; (e) HBr/AcOH (38%), r.t., 65%; (f) Zn(TfO)_2_ 1 eq, MeOH, r.t., 52%.

The multistep synthesis of the **R** receptor and **BDPA** was carried out according to Scheme 1. First, in three steps, 9-(bromo(phenyl)methylene)-9H-fluorene (2) was obtained in 83% yield starting from fluorene and benzaldehyde. The synthesis was carried out following the literature data with minor changes [15]. Then, compound **2** in the reaction with fluorene or with ethyl N-(fluorenyl)carbamate in the presence of freshly sublimed t-BuOK as a base in anhydrous conditions gave **BDPA** or compound **3** in yields of 42% and 50%, respectively. In the next step, compound **3** was introduced as an isomeric mixture due to the impossibility of separation (see SI Figures S9–S13). Deprotection of the amino group of compound **3** using 38% HBr in AcOH allowed for the chromatographic separation of the obtained isomeric amines 4a and 4b in yields of 50% and 15%, respectively (see SI Figures S13–S16). Further work was carried out only with the isomer 4a due to the higher yield obtained in comparison to its isomer. Isomer 4a was subjected to the coupling reaction with the previously prepared monoamide 5 to obtain the ion pair receptor **R** (52% yield) [28].

### 2.2. Binding Studies

Initially, the ability of the ion pair receptor **R** to complex with selected anions (Cl^−^, Br^−^, NO_2_^−^, NO_3_^−^, SO_4_^2−^, BzO^−^, and AcO^−^) or in situ generated salts was studied under the control of UV–vis spectroscopy. Due to the excessively high apparent stability constants of the complexes in acetonitrile, titrations were carried out in the presence of 1% water to create a competitive environment for ion binding (see SI Figures S26–S32). The determined apparent stability constants are presented in Table 1, which were calculated by fitting the anion-induced shifts in bathochromic absorption maxima to a 1:1 binding model using the Bindfit program. Analysis of the stability constants determined for chlorides showed an increase in binding strength by a factor of 2.7 in the presence of sodium ions and 3.1 in the presence of potassium ions. This effect indicates cooperative binding of the ion pair by the receptor **R**, which is the result of the change in the character of the alkoxy substituents of the benzocrown ether during cation binding. As expected, the stability constant was higher in the presence of 1 equiv. ion of potassium than for sodium, which is the result of a better fit of the effective macrocyclic cavity of benzo-18-crown-6 to the size of the potassium cation. For this reason, the titrations were extended to include other anions in the absence and presence of potassium ions, and in each case, the tendency for cooperative binding of the salt was preserved. For carboxylates, after adding an increasing amount of anion, a creation in a new band was observed, which was the effect of deprotonation, while in the case of sulfate the obtained data could not be fitted to established binding models, which may be the effect of the formation of complexes with a higher stoichiometry.

**Figure 2:**
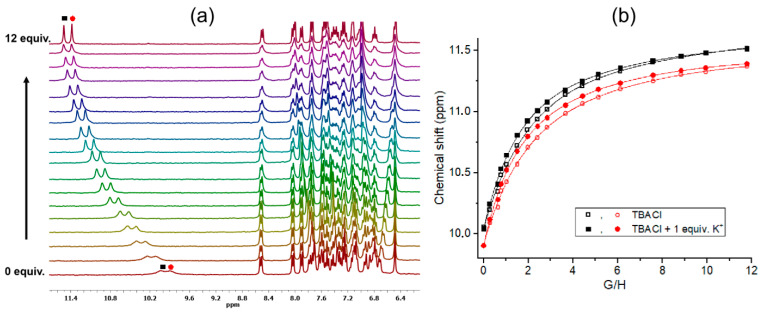
(**a**) Representative stack plots of the partial ^1^H NMR spectra of receptor **R** upon the addition of TBACl in the presence of 1 equiv. KPF_6_. (**b**) The selected binding isotherms for the titration of **R** with TBACl and TBACl in the presence 1 equiv. of KPF_6_ in DMSO-d_6_.

To support the above findings of cooperative binding of the salt by the ion pair receptor **R**, ^1^H NMR titrations were performed in DMSO-d_6_ (the solvent was chosen because of the expected range of K_a_ values suitable for NMR measurements) (Figure 2a; see SI Figures S33 and S34). We tested the halide salt, and the binding isotherms obtained from the chemical shift signals corresponding to the squaramide and aromatic protons could be fitted to a 1:1 binding model. The signals from the squaramides shifted by Δδ = 1.39 and Δδ = 1.45 ppm upon addition of 12 equivalents of the TBACl salt, whereas in the presence of potassium, they shifted by Δδ = 1.45 and Δδ = 1.49 ppm, respectively. Although the chemical shift differences are comparable, the titration in the presence of the cation resulted in more abrupt signal changes, suggesting stronger anion binding in the presence of potassium (Figure 2b). Indeed, the obtained stability constants confirm the cooperative binding of the salt formed in situ by **R** (K_TBACl_ = 218 ± 0.8% M^−1^ and K_KCl_ = 397 ± 1.4% M^−1^).

### 2.3. Polymer Studies

The next goal was to create poly(methyl methacrylate) (PMMA) systems. We started by polymerizing methyl methacrylate and **BDPA** in DMF at 100 °C; however, adding the reaction mixture to methanol did not result in precipitation. Since this attempt was unsuccessful, we turned to cyclohexanone, which was also a solvent used in the original publication [16], which in turn caused the precipitation of a white **BDPA–PMMA** precipitate upon adding the reaction mixture dropwise to methanol. This was possible due to the in situ formation of the **BDPA˙** radical, which then acted as an iniferter during the PMMA polymerization reaction. It was expected that such a process should occur in the case of the ditopic receptor **R**. To address the above challenges, the **R**˙ radical was first prepared to confirm that the **R** compound forms a stable radical. The receptor radical **R˙** was generated using freshly sublimed t-BuOK and AgNO_3_ in a mixture of DMSO and t-BuOH. The recorded EPR spectrum for the **R˙** radical was similar to other BDPA-based radicals reported in the literature [7,14], with a signal at 348 mT and no hyperfine pattern (see SI Figure S20). Finally, the **R–PMMA** polymer was prepared in a similar manner as **BDPA–PMMA**, where the ion pair receptor **R** was used as the iniferter. As inferred from gel-permeation chromatography (GPC) the polymer **R–PMMA** was characterized as M_n,GPC_ = 40.6 kDa, PDI = 1.79, and for the polymer **BDPA–PMMA**, lacking the receptor moiety in the structure, M_n,GPC_ = 61.9 kDa, PDI = 2.05.

**Figure 3:**
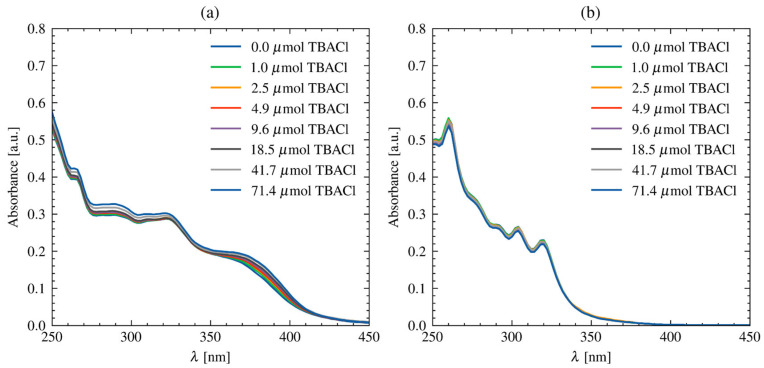
UV–vis spectra of (**a**) **R–PMMA** and (**b**) **BDPA–PMMA** upon the addition of TBACl in CHCl_3_.

Unfortunately, it is difficult to distinguish characteristic signals for **BDPA** or **R** in the ^1^H NMR spectra of the synthesized polymers, probably due to the overwhelming excess of polymer chain signals, which makes the low intensity of the iniferter signals difficult to detect by NMR spectroscopy. Similarly, the TGA profile resembles that of poly(methyl methacrylate), suggesting no significant incorporation of additional components (see SI Figures S21–S24). However, following our assumptions, the UV–vis spectra of the obtained **R–PMMA** and **BDPA–PMMA** polymers showed absorption bands in the range characteristic for their respective iniferters, where the PMMA chain itself does not show absorption. Moreover, in the case of the polymer with a built-in receptor, **R–PMMA**, the addition of TBACl to the solutions caused a change in absorbance, which indicates the preservation of the functionality of the receptor part in the polymer (Figure 3).

**Figure 4:**
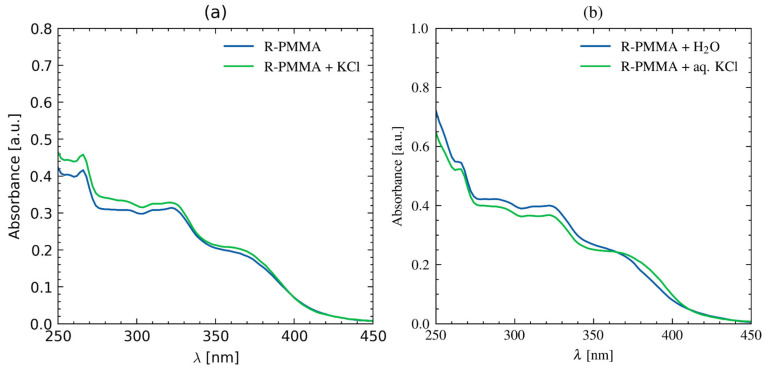
UV–vis spectra of **R–PMMA** after (**a**) the solid–liquid and (**b**) liquid–liquid extraction of KCl in CHCl_3_.

Finally, we performed solid–liquid (Figure 4a) and liquid–liquid (Figure 4b) extraction experiments using a solid and an aqueous solution of KCl and **R–PMMA** in CHCl_3_. The spectra of the latter experiment had to be normalized due to deviations from the baseline caused by the presence of water. In both cases, **R–PMMA** showed the ability to extract potassium chloride salts from the solid or aqueous solution, whereas no such effects were observed for the reference polymer **BDPA–PMMA**. This suggests that the polymer receptor **R–PMMA** is also capable of complexing salts under interfacial conditions.

## 3. Materials and Methods

### 3.1. Materials and Reagents

All reactions were performed in oven-dried glassware under a slight positive pressure of argon. ^1^H and ^13^C NMR spectra used for product characterization were recorded on a Bruker Avance 300 MHz spectrometer (Bruker, Billerica, MA, USA). Two-dimensional NMR spectra (ROESY, COSY and HSQC) were recorded on a Bruker Avance III HD 500 MHz. In each case, the spectra were calibrated to the residual solvent resonances. Chemical shifts for ^1^H NMR were reported in parts per million (ppm), calibrated to the residual solvent peak set, with coupling constants reported in Hertz (Hz). The following abbreviations were used for spin multiplicity: s = singlet, d = doublet, t = triplet, q = quadruplet, m = multiplet. Chemical shifts for ^13^C NMR spectra were reported in ppm, relative to the central line of a septet at δ = 39.52 ppm for deuteriodimethyl sulphoxide. Infrared all solvents and starting materials were purchased from commercial sources where available (Merck Europe, Fluorochem UK). The HRMS data were obtained on a Quattro LC Micromass unit (Micromass UK Ltd., Cheshire, UK). If necessary, purification of products was performed using column chromatography on silica gel (Merck Kieselgel 60, 230–400 mesh) with mixtures of solvents. Thin-layer chromatography (TLC) was performed on silica gel plates (Merck Kieselgel 60 F254). UV–vis titration experiments were performed on a Thermo Spectronic Unicam UV 500 spectrophotometer (Thermo Fisher Scientific Inc., Waltham, MA, USA) in 1% H_2_O in MeCN solution at 298 K. Proton NMR titrations were performed by adding aliquots of a putative anionic guest to a solution of the receptor in DMSO-d_6_.

### 3.2. Synthetic Details

#### 3.2.1. Preparation of Compound **1**

To a stirred solution of fluorene (3 g, 18 mmol) in dry ethanol (60 mL), benzaldehyde (1.85 mL, 18.2 mmol) and *t*-BuOK (10 g, 89 mmol) were added. The reaction was stirred at reflux under argon for 24 h. Next, the reaction mixture was washed with 40 mL of 0.5 M HCl solution and extracted with DCM (3 × 50 mL). The organic layer was collected and dried over anhydrous Na_2_SO_4_. Solvent was evaporated and the residue was purified using column chromatography, eluting with pentane. The collected product was further purified by crystallization from hexane and dried under reduced pressure to remove the residual solvent, obtaining the product 1 as a white crystalline solid. Yield 3.81 g (15 mmol, 83%). HRMS (ESI): calcd for C_20_H_15_ [M + H]^+^ 255.11683, found 255.11678. ^1^H NMR (300 MHz, DMSO-d_6_, δ ppm): 8.08–7.95 (d, 1H), 7.93 (s, 1H), 7.90–7.80 (m, 2H), 7.65–7.57 (m, 2H), 7.55–7.30 (m, 7H), 7.17–7.03 (t, 1H). ^13^C NMR (75 MHz, DMSO-d_6_, δ ppm): 141.2, 139.4, 138.8, 136.8, 136.2, 135.9, 129.5, 129.3, 129.1, 128.9, 128.8, 128.8, 127.7, 127.3, 124.1, 121.3, 120.7, 120.3.

#### 3.2.2. Preparation of 9-Bromo-9-[bromo-(phenyl)methyl]-9H-fluorene

To a stirred solution of compound **1** (3.81 g, 15 mmol) in CCl_4_ (15 mL), Br_2_ (0.93 mL, 18 mmol) was added dropwise and the reaction was stirred at room temperature for 20 min. After that time, the reaction mixture was washed with 25 mL of 1M NaHSO_3_ solution and extracted with DCM (2 × 20 mL). The collected organic layer was dried over anhydrous Na_2_SO_4_ and the solvent was removed and dried under reduced pressure to remove the residual solvent to afford 5.19 g (12.5 mmol, 83%) of pure product as a crystalline white solid. Measured spectra correspond to the literature data [15]. ^1^H NMR (300 MHz, DMSO-d_6_, δ ppm): 8.35–815 (m, 1H), 7.90–7.75 (m, 1H), 7.75–7.65 (m, 1H), 7.65–7.55 (m, 1H), 7.55–7.40 (m, 2H), 7.35–7.15 (m, 2H), 7.10–6.80 (m, 5H), 6.50 (s, 1H). ^13^C NMR (75 MHz, DMSO-d_6_, δ ppm): 145.5, 145.2, 139.5, 138.5, 137.1, 130.6, 130.0, 129.3, 128.8, 128.4, 127.7, 126.9, 126.4, 121.0, 120.8, 67.37, 62.0.

#### 3.2.3. Preparation of Compound **2**

To a stirred solution of 9-bromo-9-[bromo-(phenyl)methyl]-9H-fluorene (5.19 g, 12.5 mmol) in dry ethanol (50 mL), NaOH (2 g, 50 mmol) was added. The reaction was heated to a reflux for 2 h. The reaction mixture was cooled to room temperature, washed with 0.5 M HCl solution (50 mL) and extracted with DCM (3 × 50 mL). Organic layers were collected and dried over anhydrous Na_2_SO_4_. The solvent was removed, and the residue was purified using column chromatography, eluting with pentane. The collected product was further purified by crystallization from ethanol, yielding 3.5 g (10.5 mmol, 84%) of pure product **2** as a bright yellow crystalline solid. Measured spectra matched literature data [15]. ^1^H NMR (300 MHz, DMSO-d_6_, δ ppm): 8.85–8.70 (d, 1H), 8.00–7.90 (d, 1H), 7.90–7.75 (d, 1H), 7.70–7.35 (m, 7H), 7.32–7.20 (t, 1H), 6.97–6.80 (t, 1H), 6.15-6.02 (d, 1H). ^13^C NMR (75 MHz, DMSO-d_6_, δ ppm): 142.6, 141.1, 139.7, 137.8, 137.4, 135.6, 130.1, 130.1, 130.0, 129.1, 128.6, 127.9, 127.5, 126.0, 125.2, 124.5, 120.7, 120.4.

#### 3.2.4. Preparation of Ethyl N-(Fluorenyl)carbamate

To a stirred solution of 2-aminofluorene (2 g, 11 mmol) and DIPEA (2.7 mL, 15.5 mmol) in DMF (30 mL), ethyl chloroformate (1.21 mL, 12.7 mmol) was added dropwise. The reaction was carried out at room temperature under argon for 24 h. The reaction mixture was washed with 0.5 M HCl solution (30 mL) and extracted with DCM (2 × 30 mL). Combined organic layers were washed with water (30 mL) and dried over anhydrous Na_2_SO_4_. Solvent removal yielded 2.49 g (9.8 mmol, 89%) of ethyl N-(fluorenyl)carbamate as an off-white crystalline solid. HRMS (ESI): calcd for C_16_H_15_NO_2_Na [M + Na] ^+^ 276.0995, found 276.0998. ^1^H NMR (300 MHz, DMSO-d_6_, δ ppm): 9.71 (s, 1H), 7.86–7.68 (m, 3H), 7.58–7.50 (d, 1H), 7.50–7.42 (d, 1H), 7.34 (t, 1H), 7.24 (t, 1H), 4.24–4.05 (q, 2H), 3.88 (s, 2H), 1.26 (t, 3H). ^13^C NMR (75 MHz, DMSO-d_6_, δ ppm): 154.1, 144.3, 143.1, 141.5, 138.8, 136.0, 127.1, 126.4, 125.4, 120.6, 119.8, 117.5, 115.3, 60.6, 36.9, 15.0.

#### 3.2.5. Preparation of BDPA

To a dry flask under argon atmosphere, compound **2** (700 g, 2.1 mmol), fluorene (340 mg, 2.1 mmol) and freshly sublimed t-BuOK (1.18 g, 10.5 mmol) were added. Next, the reaction vessel was subjected to 3 vacuum-argon cycles, placed into a cooling bath and the reactants were dissolved in dry THF (20 mL). The reaction was carried out for 24 h at room temperature. The reaction mixture was then washed with 0.5 M HCl solution (20 mL) and extracted twice with DCM (2 × 20 mL). Combined organic layers were dried over anhydrous Na_2_SO_4_ and then the solvent was removed. The crude product was purified using column chromatography eluting with a gradient from pure pentane to pentane: DCM (9:1), with a yield of 370 mg (0.89 mmol, 42%) of BDPA as a crystalline orange solid. Measured spectra correspond to the literature data [15].

#### 3.2.6. Preparation of Compound **3**

To a dry flask under argon atmosphere compound **2** (3.2 g, 9.6 mmol), ethyl N-(fluorenyl)carbamate (2.44 g, 9.6 mmol) and freshly sublimed t-BuOK (4 g, 35.6 mmol) were added. Next, the reaction vessel was subjected to 3 vacuum-argon cycles, placed into a cooling bath and the reactants were dissolved in anhydrous THF (60 mL). The reaction was carried out for 24 h at room temperature. The reaction mixture was then washed with 0.5 M HCl solution (50 mL) and extracted twice with DCM (2 × 50 mL). Combined organic layers were dried over anhydrous Na_2_SO_4,_ and then the solvent was removed. The crude product was purified using column chromatography eluting with a gradient from pentane: Et_2_O (9:1 to 8:2), yielding 2.4 g (4.8 mmol, 50%) of compound **3** as a crystalline yellow solid. Two-dimensional NMR analysis performed on the reaction product showed the existence of two isomers: 3a and 3b (for more details on ^1^H and ^13^C NMR spectra, see SI Figures S9–S13). HRMS (ESI): calcd for C_36_H_27_NO_2_Na [M + Na]^+^ 528.1934, found 528.1941.

#### 3.2.7. Preparation of Compound **4**

Compound **3** (2.4 g, 4.75 mmol) was dissolved in 38% HBr in AcOH (12 mL). The reaction was carried out for 24 h at room temperature. Next, a saturated NaHCO_3_ solution was slowly added to the reaction mixture until it reached an alkaline pH. The resulting mixture was extracted with DCM (3 × 50 mL) and the combined organic layers were dried over anhydrous MgSO_4_. The solvent was removed, and the residue was purified using column chromatography eluting with a pentane: Et_2_O mixture (6:4), which yielded 1 g (2.3 mmol) of compound **4a** and 300 mg (0.7 mmol) of compound **4b** as yellow solids with a combined reaction yield of 65%.

Compound **4b**: HRMS (ESI): calcd for C_33_H_24_N [M + H]^+^ 434.1903, found 434.1907. ^1^H NMR (300 MHz, DMSO-d_6_, δ ppm): 7.84–7.75 (d, 3H), 7.65–7.55 (m, 4H), 7.33 (quint, 4H), 7.11–6.99 (m, 4H), 6.74–6.69 (d, 1H), 6.64–6.52 (m, 3H), 6.42 (s, 1H), 5.57–5.49 (d, 1H), 5.33 (s, 2H). ^13^C NMR (75 MHz, DMSO-d_6_, δ ppm): 149.6, 144.4, 144.0, 141.9, 141.3, 140.0, 139.1, 137.8, 136.3, 129.7, 128.4, 128.2, 128.0, 127.7, 127.6, 126.3, 125.0, 124.4, 121.2, 120.6, 117.9, 114.5, 111.3, 52.4.

Compound **4a**: HRMS (ESI): calcd for C_33_H_23_NNa [M + Na]^+^ 456.1722, found 456.1727. ^1^H NMR (300 MHz, DMSO-d_6_, δ ppm): 8.48–8.41 (d, 1H), 8.04–7.98 (d, 1H), 7.91–7.85 (d, 1H), 7.52–7.41 (m, 5H), 7.23–7.05 (m, 6H), 6.87–6.76 (m, 3H), 6.63–6.57 (d, 2H), 6.32 (s, 1H), 5.72–5.64 (d, 1H), 5.26 (s, 2H). ^13^C NMR (75 MHz, DMSO-d_6_, δ ppm): 149.2, 146.6, 145.8, 143.0, 142.9, 140.9, 139.8, 139.1, 138.7, 138.4, 135.4, 130.4, 129.3, 128.7, 128.3, 128.2, 128.0, 127.9, 127.6, 126.9, 126.1, 125.7, 125.2, 124.8, 121.3, 120.6, 119.9, 118.6, 114.1, 110.9, 52.5.

#### 3.2.8. Preparation of Compound **5**

4-Aminobenzo-18-crown-6 was synthesized as described in the literature [19]. To a solution of 4-aminobenzo [18] crown-6 (1026 mg, 3.1 mmol) in MeOH (25 mL), dimethyl squarate (450 mg, 3.2 mmol) was added. The reaction was carried out for 24 h at room temperature. The resulting suspension was centrifuged to yield an off-white solid, which was further washed with MeOH and dried in vacuo to yield 1020 mg (2.3 mmol, 74%) of monoamide 5 as an off-white solid. Measured spectra correspond to the literature data [19].

#### 3.2.9. Preparation of Receptor **R**

To a stirred solution of compound **4a** (500 mg, 1.15 mmol) and Zn(TfO)_2_ (420 mg, 1.15 mmol) in MeOH (25 mL), compound **5** (505 mg, 1.15 mmol) was added. The reaction was carried out for 24 h at room temperature. The resulting suspension was centrifuged to yield a yellowish solid that was further washed with MeOH. The centrifuged precipitate was dissolved in DCM (25 mL) and the resulting solution was extracted with water (25 mL). The water layer was extracted once with DCM (1 × 25 mL) and the combined organic layers were dried over anhydrous MgSO_4_ and then the solvent was removed. The crude product was recrystallized from MeOH to yield 508 mg (0.61 mmol, 52%) of compound **R** as a greenish solid. HRMS (ESI): calcd for C_33_H_24_N [M + H]^+^ 434.1903, found 434.1907. ^1^H NMR (300 MHz, DMSO-d_6_, δ ppm): 10.17 (s, 1H), 10.02 (s, 1H), 8.55–8.44 (d, 1H), 8.05–7.98 (d, 1H), 7.90–7.86 (d, 1H), 7.79–7.69 (m, 3H), 7.62–7.54 (m, 2H), 7.50 (t, 1H), 7.41 (t, 1H), 7.35–7.29 (m, 2H), 7.23 (t, 2H), 7.14–7.03 (m, 3H), 6.94–6.84 (m, 2H), 6.81–6.74 (m, 2H), 6.71–6.65 (d, 1H), 6.47 (s, 1H), 6.70–6.62 (d, 1H), 4.09–4.00 (m, 4H), 3.78–3.71 (m, 4H), 3.60–3.51 (m, 12H). ^13^C NMR (75 MHz, DMSO-d_6_, δ ppm): 182.1, 181.3, 165.9, 148.7, 145.8, 145.3, 144.6, 143.9, 141.6, 141.0, 139.8, 138.8, 138.6, 138.5, 138.2, 137.1, 135.8, 132.6, 128.9, 128.6, 128.3, 128.2, 128.0, 127.0, 126.2, 125.8, 125.2, 120.7, 120.2, 120.0, 116.0, 113.5, 104.8, 70.0, 69.0, 68.2, 52.8, 40.8, 40.5, 40.3, 40.0, 39.7, 39.4, 39.1.

#### 3.2.10. Preparation of Radical **R˙**

To a solution of compound **R** (30 mg, 36 µmol) in DMSO:t-BuOH (4:1 *v*/*v*, 25 mL), freshly sublimed t-BuOK (80 mg, 713 µmol) was added. The reaction was carried out for 30 min at room temperature, after which AgNO_3_ (97 mg, 571 µmol) dissolved in 1 mL of H_2_O was added, which resulted in a color change from blue to deep red. After about 1 min, the reaction mixture was washed with 0.1 M HCl (200 mL) and extracted with diethyl ether (3 × 50 mL). Combined organic layers were dried over anhydrous MgSO_4_ and then the solvent was removed. The crude product was purified using column chromatography eluting with dichloromethane, which yielded 1.52 mg (1.81 µmol, 5%) of radical **R˙** as a dark red solid (EPR spectrum of compound **R˙** in DMSO; see SI Figure S20). HRMS (ESI): calcd for C_53_H_45_N_2_O_8_ [M]^+^ 837.3170, found 837.3147.

#### 3.2.11. Preparation of **BDPA–PMMA**

A solution of BDPA (21 mg, 0.05 mmol) and methyl methacrylate (500 μL, 5 mmol) in cyclohexanone (1 mL) was subjected to 3 vacuum-argon cycles and left to react overnight at 100 °C. The reaction mixture was then diluted with THF (1.5 mL) and added dropwise to MeOH (20 mL). The precipitated polymer was then redissolved in THF (3 mL), reprecipitated in MeOH (20 mL) and dried in vacuum to obtain **BDPA–PMMA**.

#### 3.2.12. Preparation of **R–PMMA**

A solution of compound **R** (42 mg, 0.05 mmol) and methyl methacrylate (500 μL, 5 mmol) in cyclohexanone (1 mL) was subjected to 3 vacuum-argon cycles and left to react overnight at 100 °C. The reaction mixture was then diluted with THF (1.5 mL) and added dropwise to MeOH (20 mL). The precipitated polymer was then redissolved in THF (3 mL), reprecipitated in MeOH (20 mL) and dried in vacuum to obtain **R–PMMA**.

## 4. Conclusions

In summary, an effective iniferter was prepared as the ion pair receptor R, based on squaramide and containing a BDPA unit. Complexing properties towards selected anions and cooperative binding of salt by the receptor R were demonstrated using the UV–vis titration technique and an independent ^1^H NMR titration technique. Then, the R–PMMA and BDPA–PMMA polymers were obtained, where the receptor R and BDPA acted as the iniferters, respectively. The obtained polymers were characterized by GPC, revealing a weight distribution characteristic of an uncontrolled (free-radical) polymerization process. UV–vis spectroscopy confirmed the presence of both BDPA and R structural units in the obtained polymers. The incorporation of the receptor R into the polymer structure preserved the functional properties of the receptor moiety, namely the ability to interact with both anions and cations. This functional behavior was not observed in the BDPA–PMMA reference material. The functionality of R–PMMA was further confirmed through extraction studies, which demonstrated its capability to extract salts from both solid and aqueous phases into the organic phase.

## Figures and Tables

**Table 1:** Association constants for interactions of receptor **R** with selected anions ^a^.

K_a_/1000 M^−1^ ± Error	TBA^+^	Na^+^	K^+^
**Cl^−^**	20.7 ± 0.8%	56.3 ± 1.6%	63.2 ± 1.4%
**Br^−^**	18.0 ± 2.4%	^b^	29.6 ± 2.9%
**NO_3_^−^**	^c^	^b^	^c^
**NO_2_^−^**	10.3 ± 1.3%	^b^	14.1 ± 1.4%
**BzO^−^**	^d^	^b^	^d^
**AcO^−^**	^d^	^b^	^d^
**SO_4_^2−^**	^e^	^b^	^e^

^a^ UV–vis, solvent 1% H_2_O in MeCN, temperature 293 K, [**R**] = 2.0 × 10^−5^ M; anions added as TBA salts [TBAX] ∼ 1 × 10^−3^ M; M^−1^, errors < 5%. ^b^ Not determined. ^c^ Low changes in the UV–vis spectrum upon titration to determine K_a_. ^d^ Deprotonation. ^e^ The obtained data could not be fitted to an appropriate binding model.

## Data Availability

The original contributions presented in this study are included in the article/Supplementary Material. Further inquiries can be directed to the corresponding author(s).

## Supplementary Materials

The following supporting information can be downloaded at: https://www.mdpi.com/article/10.3390/molecules30163362/s1.
