# Supplementary material for: From Binding to Building: A Squaramide-Based Ion Pair Receptor as an Iniferter for Functional Polymer Synthesis
Source: Molecules. 2025 Aug 13;30(16):3362. doi: 10.3390/molecules30163362 (PMC12388569; doi:10.3390/molecules30163362)
Supplement: Supplementary file 1 [file molecules-30-03362-s001.zip › molecules-3771758-supplementary.pdf]

From Binding to Building: A Squaramide-Based Ion Pair Receptor as an Iniferter for Functional Polymer Synthesis

Mikołaj Prokopski, Marta Zaleskaya-Hernik, Wojciech Witkowski, Piotr Garbacz, Jan Romański

Table of Contents

1. **General information** **S1**
2. **Spectral data** **S2**
3. **UV-Vis titration experiments** **S16**
4. **NMR titration experiments** **S20**
5. **Solid-liquid extraction experiments** **S21**
6. **Liquid-liquid extraction experiments** **S22**
7. **References** **S22**

1. General information

Unless specifically indicated, all chemicals and reagents used in this study were purchased from commercial sources and used as received. If necessary, purification of products was performed using column chromatography on silica gel (Merck Kieselgel 60, 230- 400 mesh). Thin-layer chromatography (TLC) was performed on silica gel plates (Merck Kieselgel 60 F254). ^{1}H and ^{13}C NMR spectra used in the characterization of products were recorded on Bruker Avance 300 MHz spectrometer. Two-dimensional NMR spectra (ROESY, COSY and HSQC) were recorded on a Bruker Avance III HD 500 MHz. In each case, the spectra were calibrated to the residual solvent resonances. The HRMS data were obtained on a Quattro LC Micromass unit.

2. **Spectral data**

Compound 1.

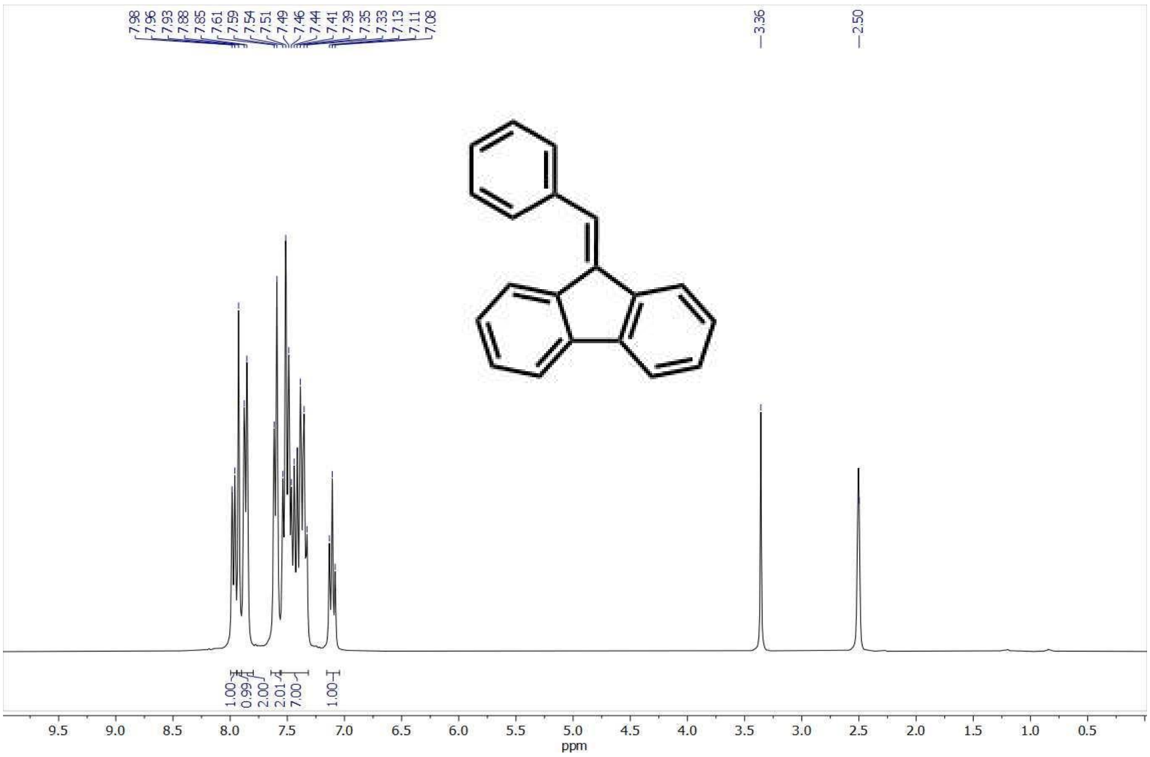

Figure S1. ^{1}H NMR spectrum of compound **1** in DMSO-d_6 .

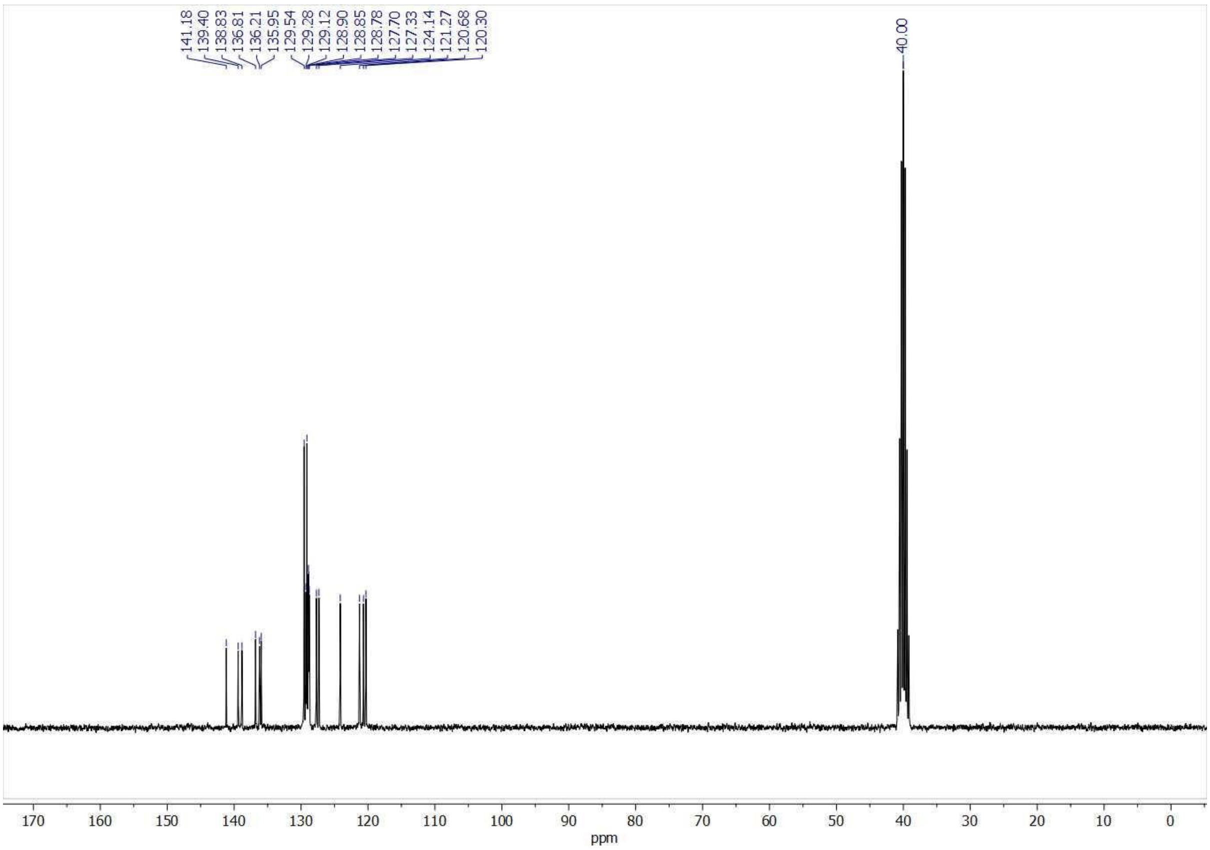

Figure S2. ^{13}C NMR spectrum of compound **1** in DMSO-d_6.

9-bromo-9-[bromo-(phenyl)methyl]-9H-fluorene.

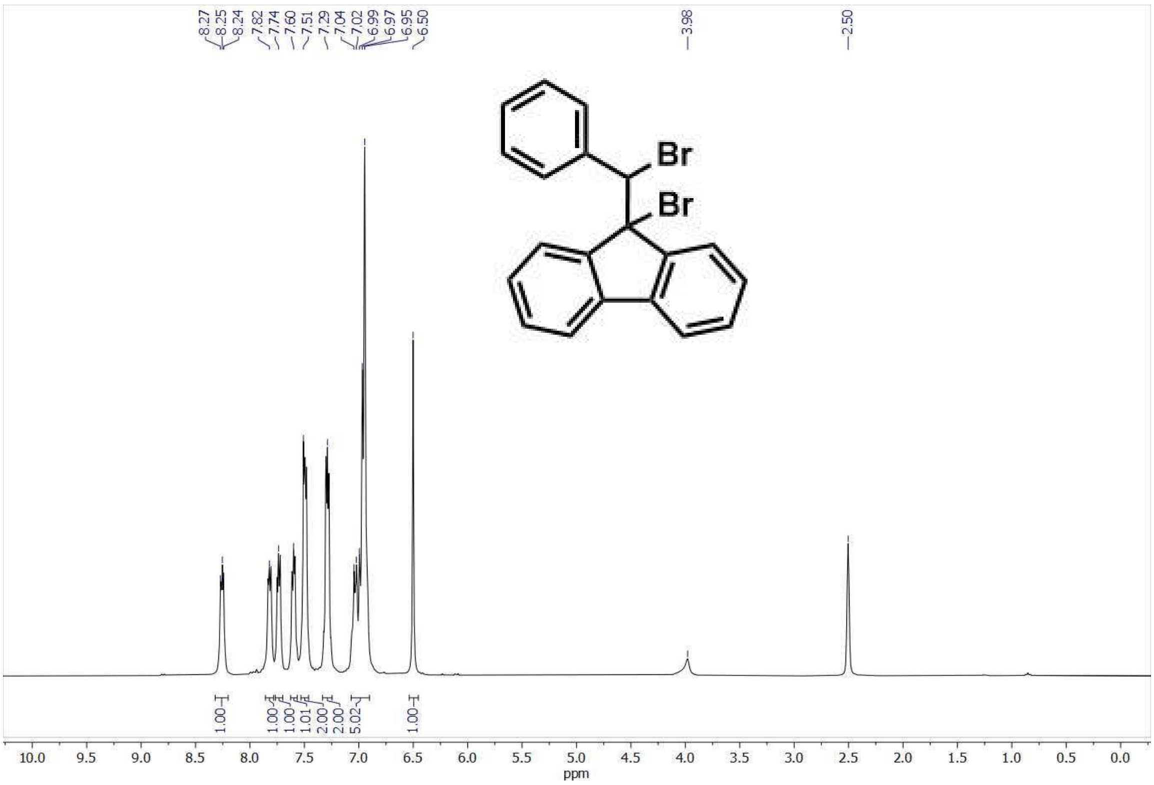

Figure S3. ^{1}H NMR spectrum of 9-bromo-9-[bromo-(phenyl)methyl]-9H-fluorene in DMSO-d_6.

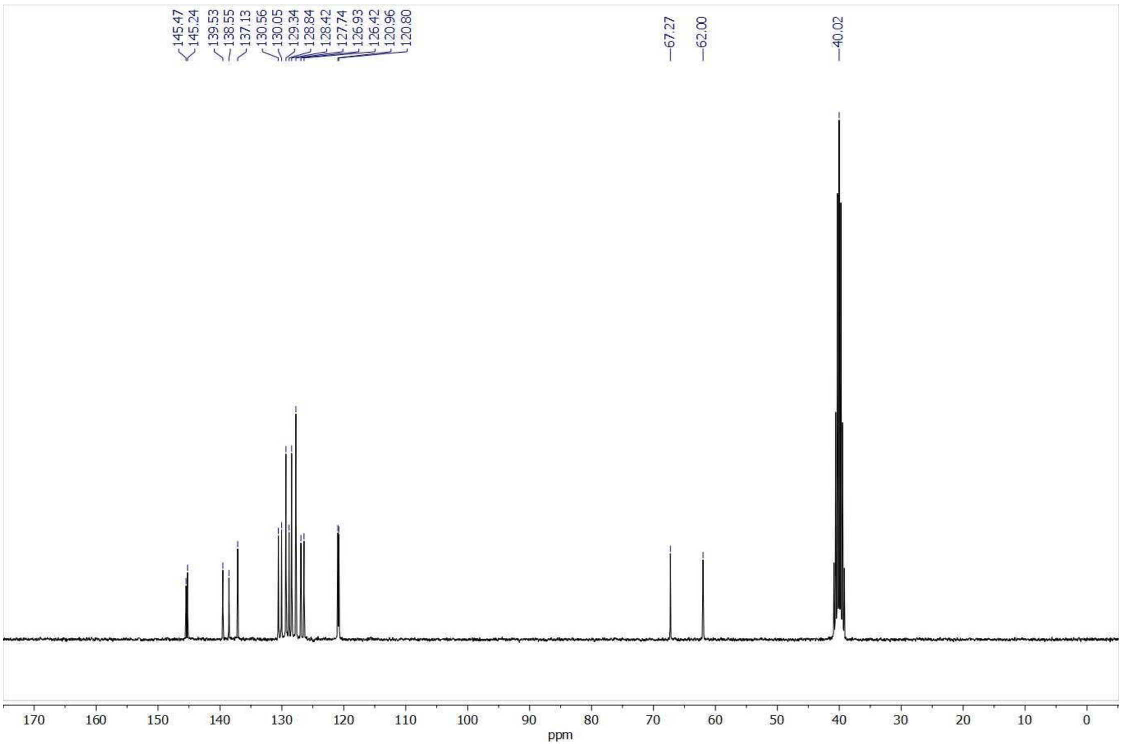

Figure S4. ^{13}C NMR spectrum of 9-bromo-9-[bromo-(phenyl)methyl]-9H-fluorene in DMSO-d_6.

Compound 2.

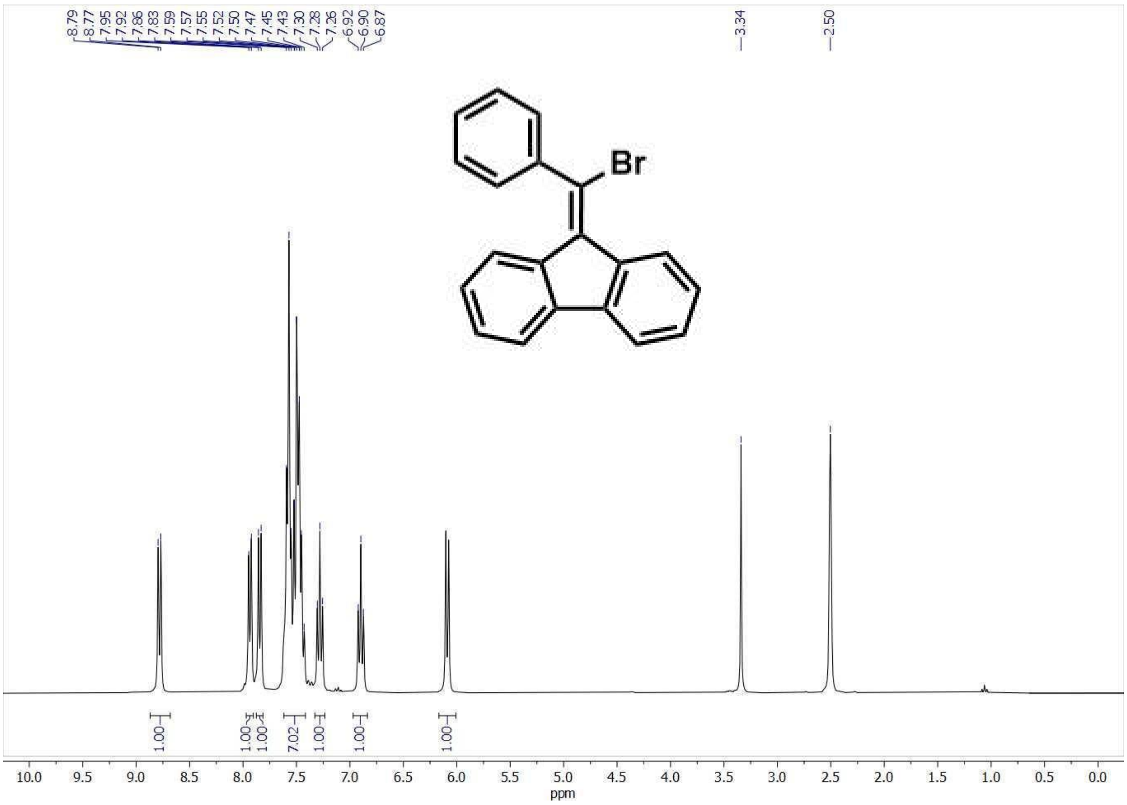

Figure S5. ^{1}H NMR spectrum of compound **2** in DMSO-d_6.

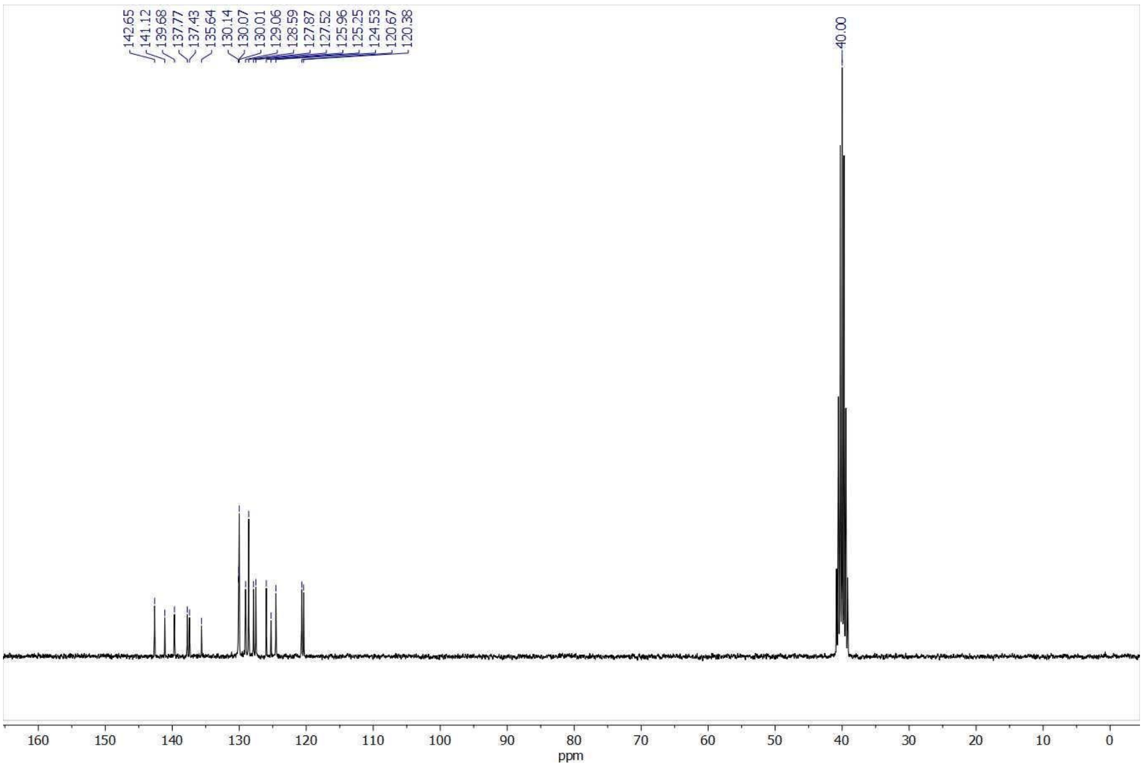

Figure S6. ^{13}C NMR spectrum of compound **2** in DMSO-d_6.

Ethyl N-(fluorenyl)carbamate.

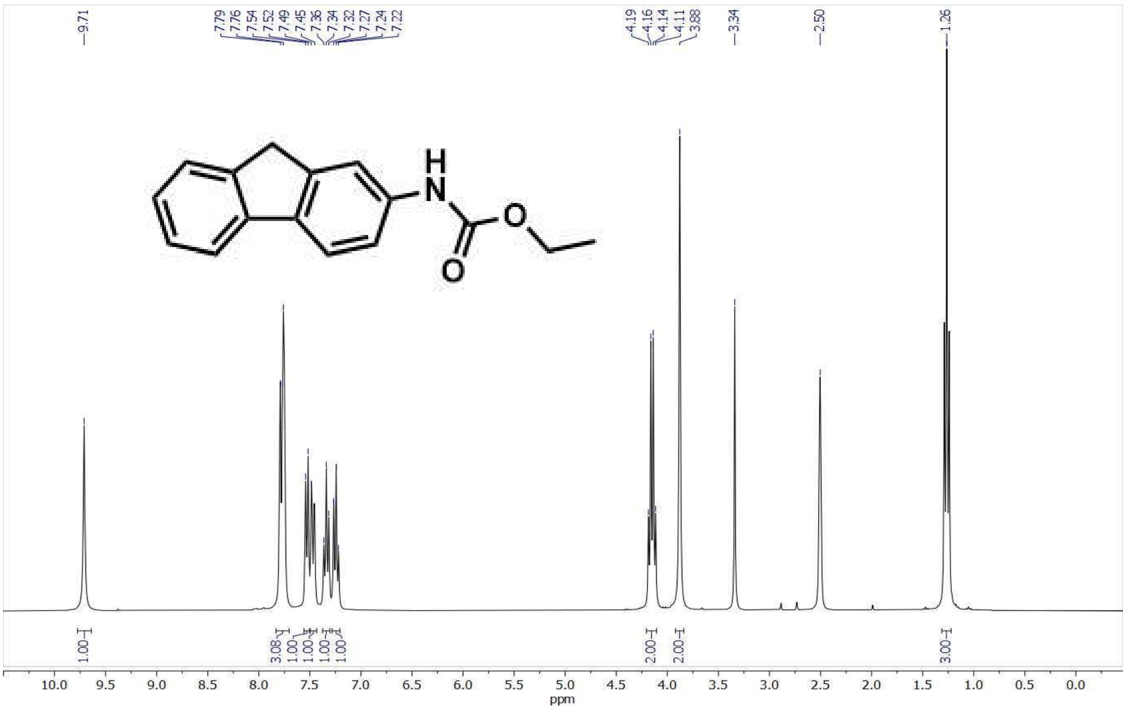

Figure S7. ^{1}H NMR spectrum of ethyl N-(fluorenyl)carbamate in DMSO-d_6.

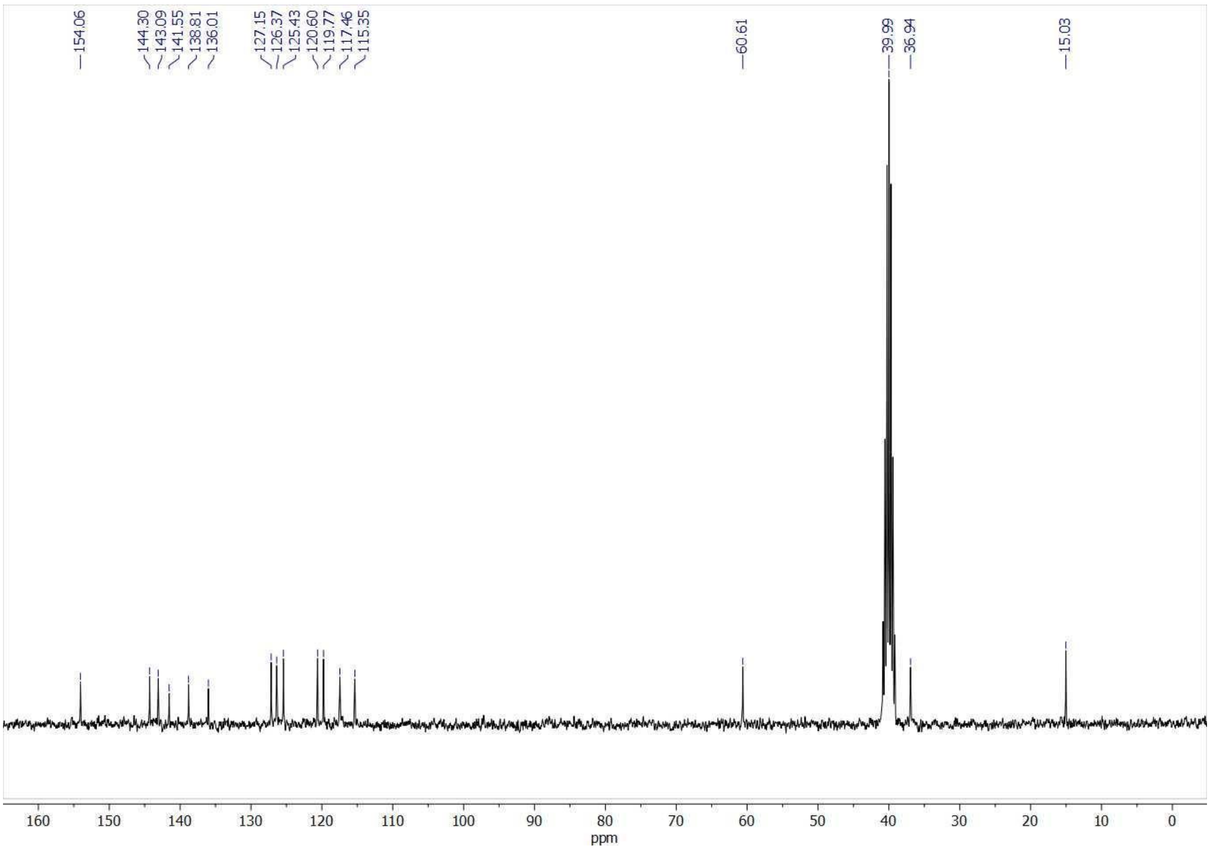

Figure S8. ^{13}C NMR spectrum of ethyl N-(fluorenyl)carbamate in DMSO-d_6.

Compound 3.

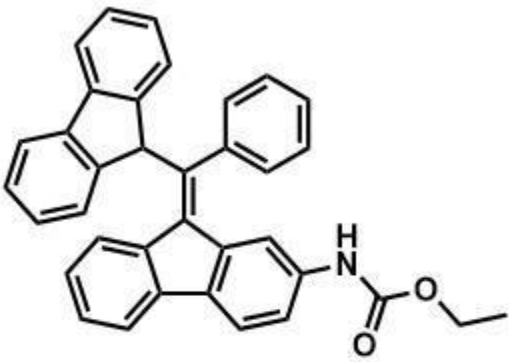

To a dry flask under argon atmosphere compound **2** (3.2 g, 9.6 mmol), ethyl N-(fluorenyl)carbamate (2.44 g, 9.6 mmol) and freshly sublimed t-BuOK (4 g, 35.6 mmol) were added. Next, the reaction vessel was subjected to 3 vacuum-argon cycles, placed into a cooling bath and the reactants were dissolved in anhydrous THF (60 mL). The reaction was carried out for 24 h at room temperature. The reaction mixture was then washed with 0.5 M HCl solution (50 mL) and extracted twice with DCM (2 x 50 mL). Combined organic layers were dried over anhydrous Na_2SO_4, and then the solvent was removed. The crude product was purified using column chromatography eluting with a gradient from pentane: Et_2O (9:1 to 8:2), yielding 2.4 g (4.8 mmol, 50%) of compound 3 as a crystalline yellow solid. 2D NMR analysis performed on the reaction product showed the existence of two isomers: 3a and 3b (For more details on ^{1}H and ^{13}C NMR spectra, see SI Figures S10 - S13). HRMS (ESI): calcd for $C_{36}H_{27}NO_2Na$ $[M + Na]^+$ 528.19340, found 528.19414.

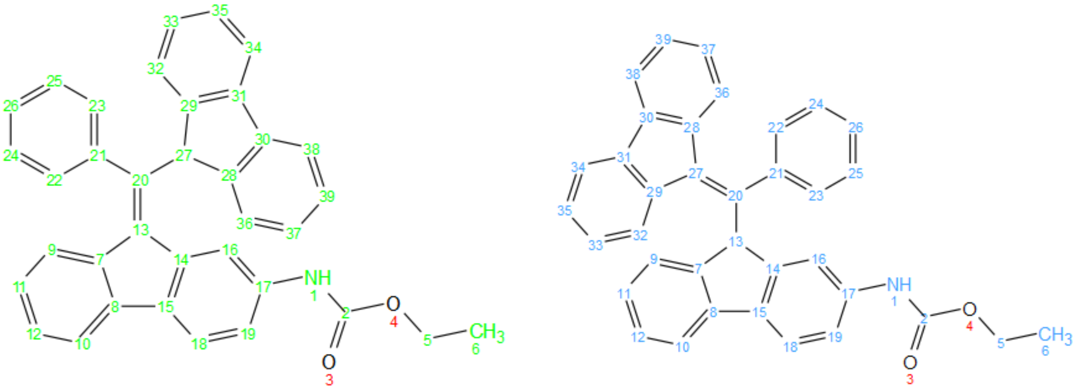

Figure S9. NMR assignments of compounds **3b** (green) and **3a** (blue).

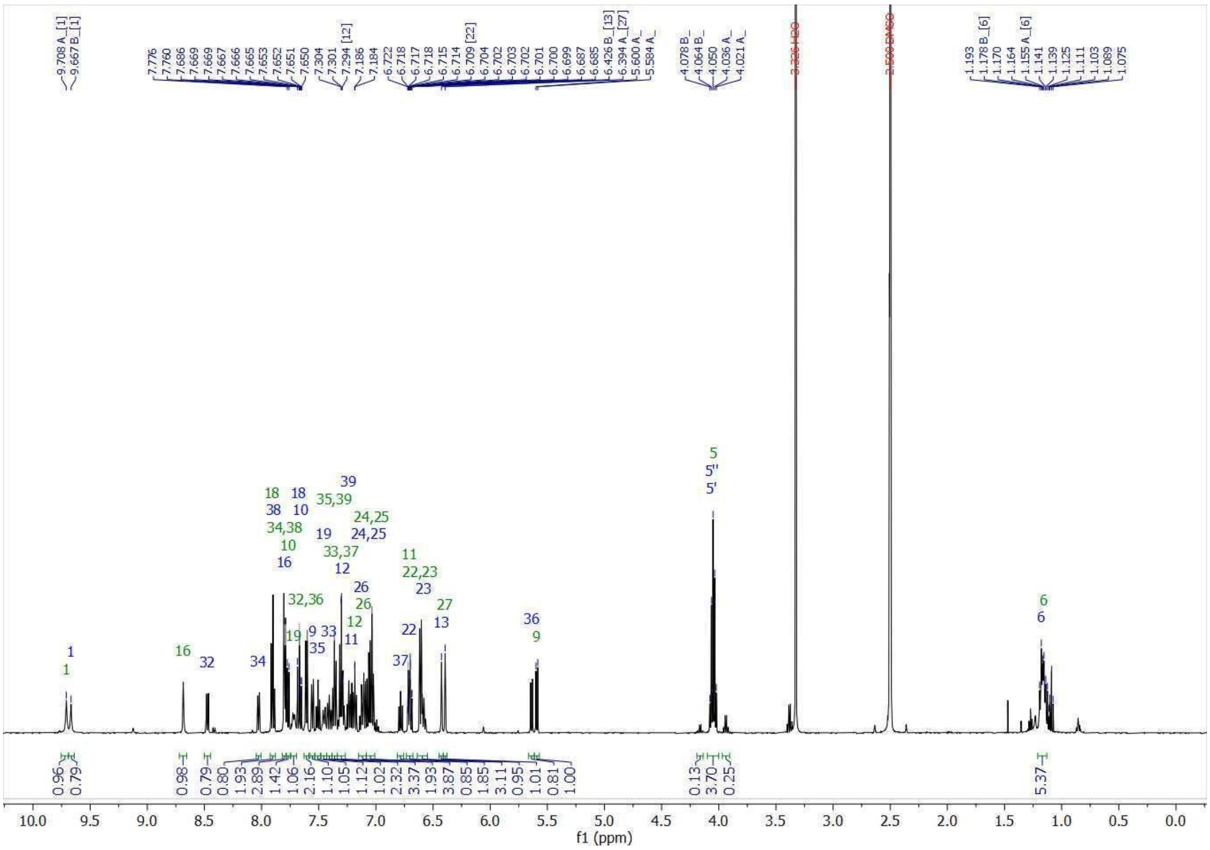

Figure S10. ^{1}H NMR spectrum of compounds **3b** and **3a** in DMSO-d_6.

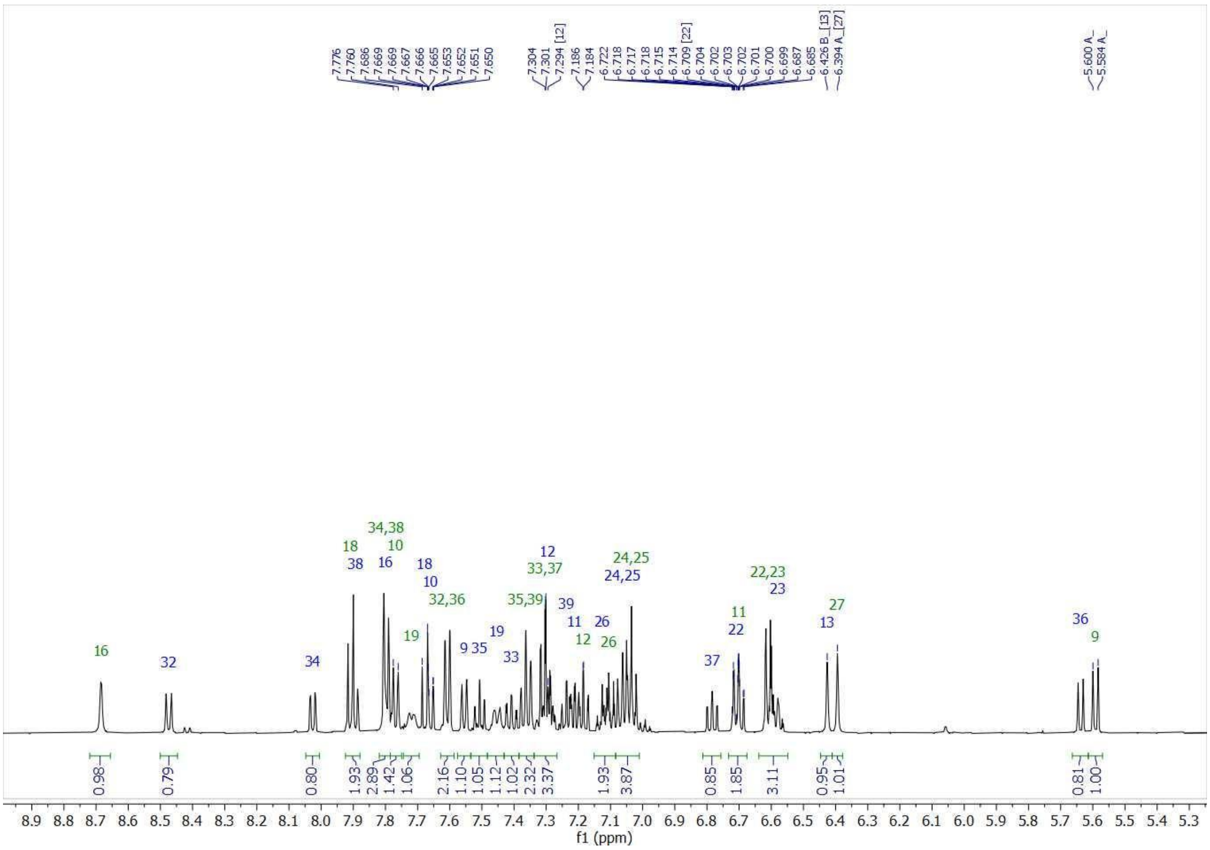

Figure S11. ^{1}H NMR spectrum of compounds **3b** and **3a** in DMSO-d_6 (close-up of aromatic region).

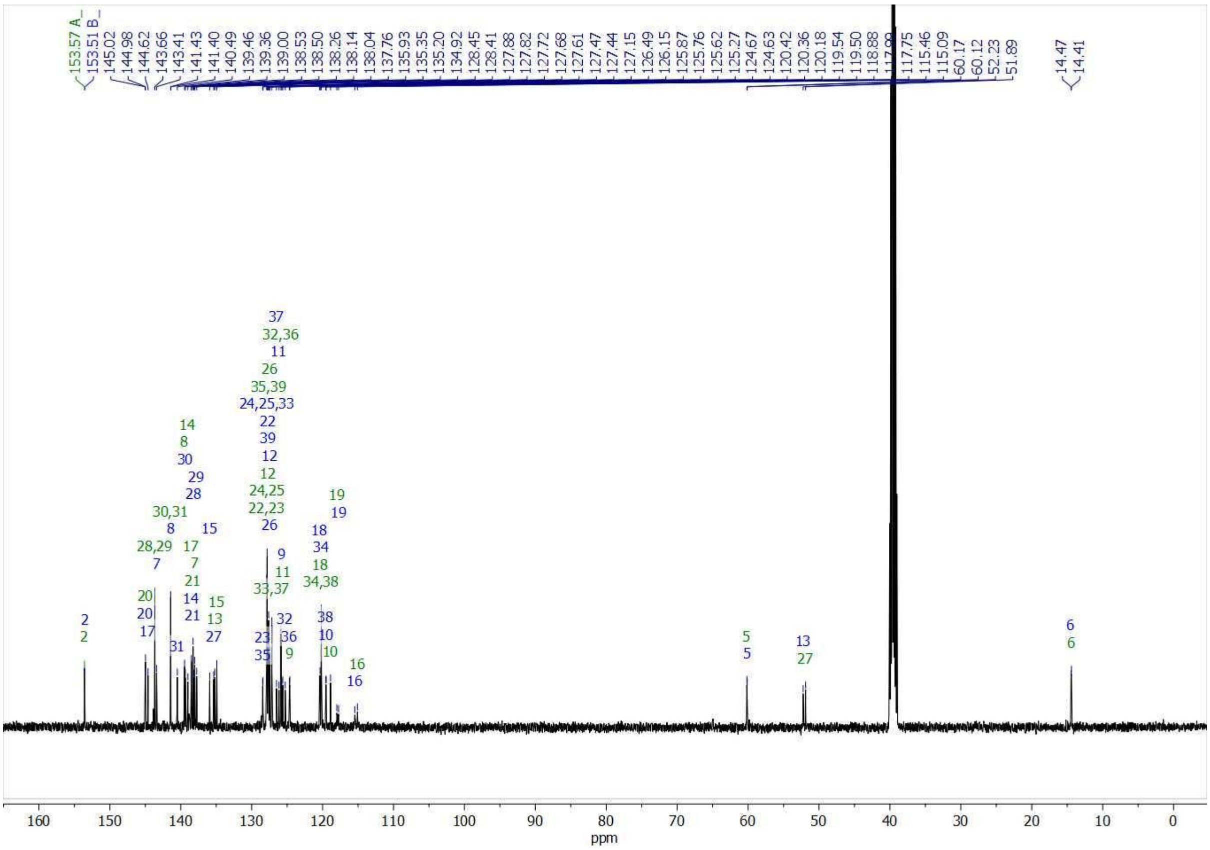

Figure S12. ^{13}C NMR spectrum of compound **3b** and **3a** in DMSO-d$_6$.

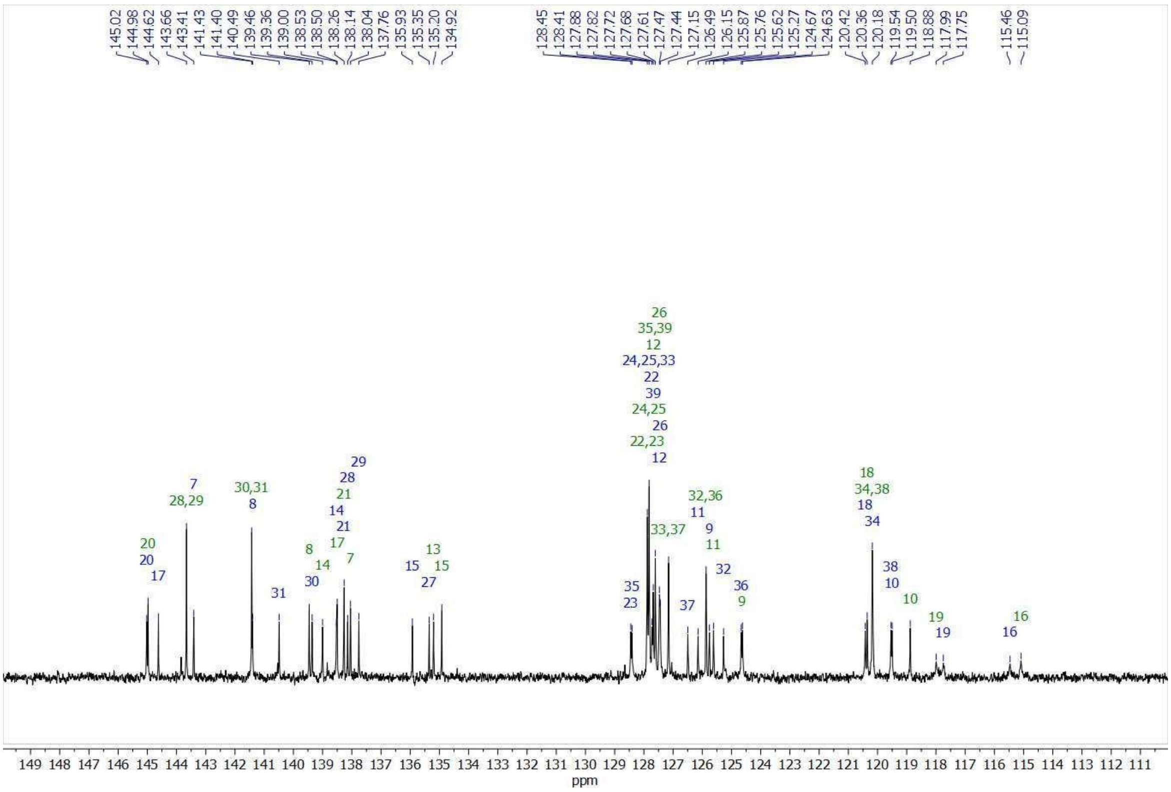

Figure S13. ^{13}C NMR spectrum of compounds **3b** and **3a** in DMSO-d$_6$ (close-up of the densest region).

Compounds 4a, 4b.

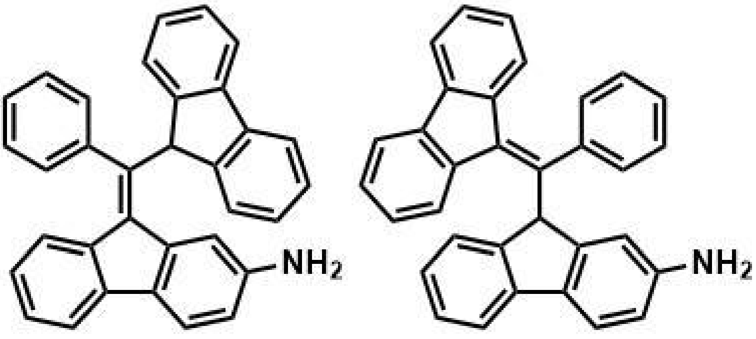

4b 4a

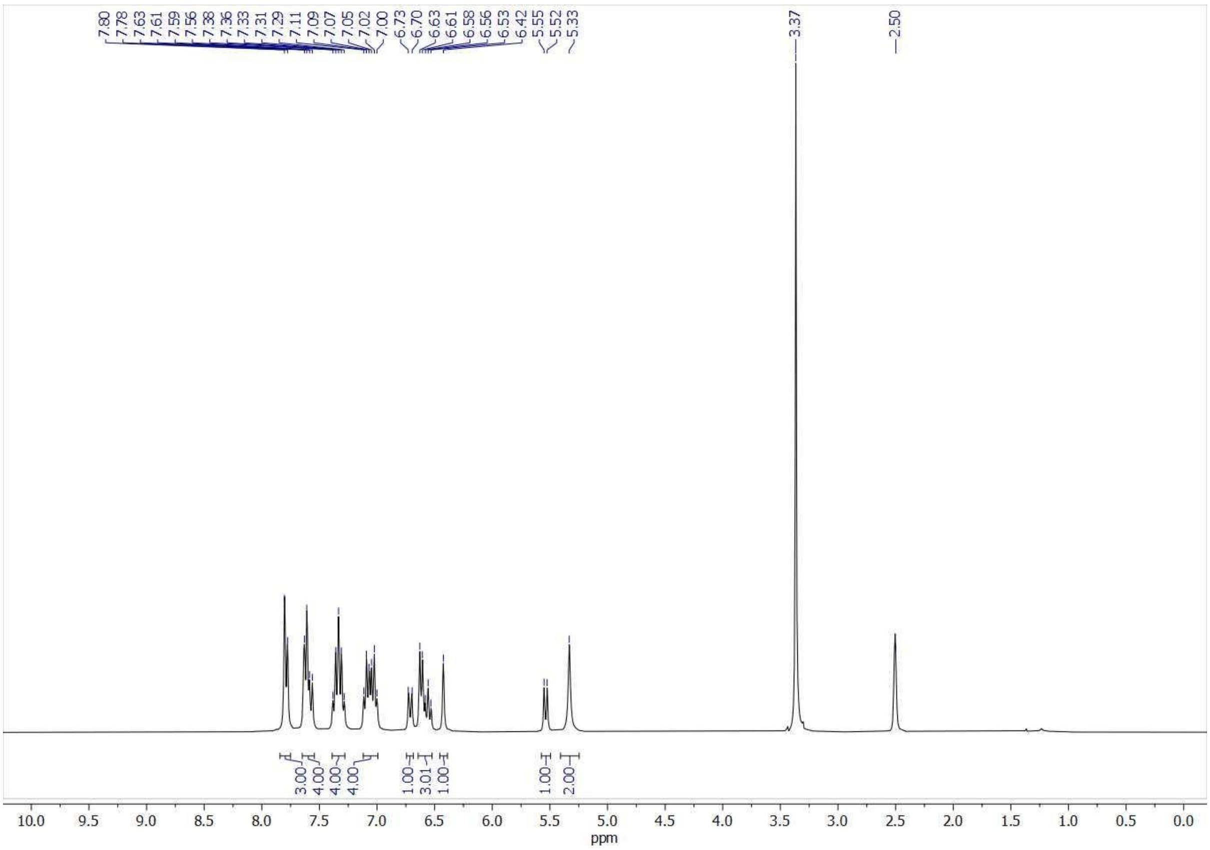

Figure S14. ^{1}H NMR spectrum of compound **4b** in DMSO-d_6.

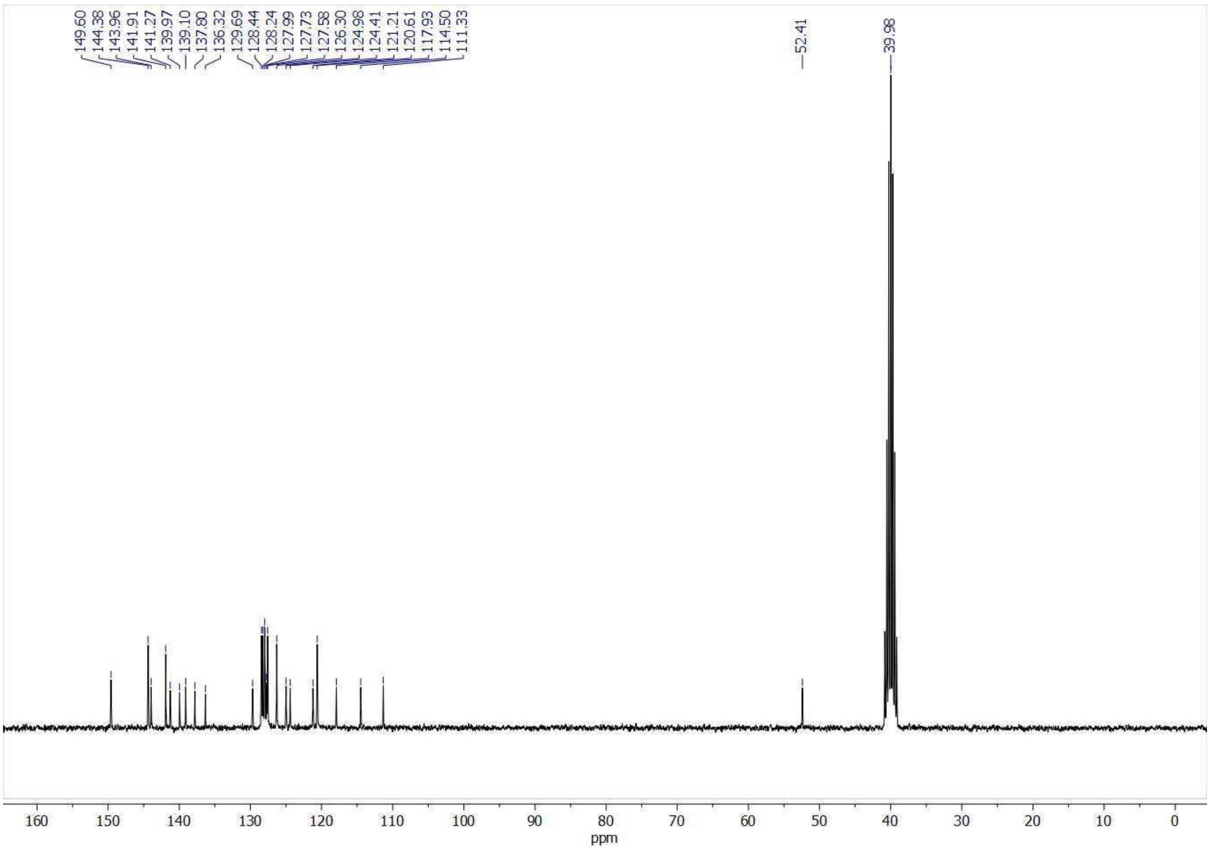

Figure S15. ^{13}C NMR spectrum of compound **4b** in DMSO-d_6.

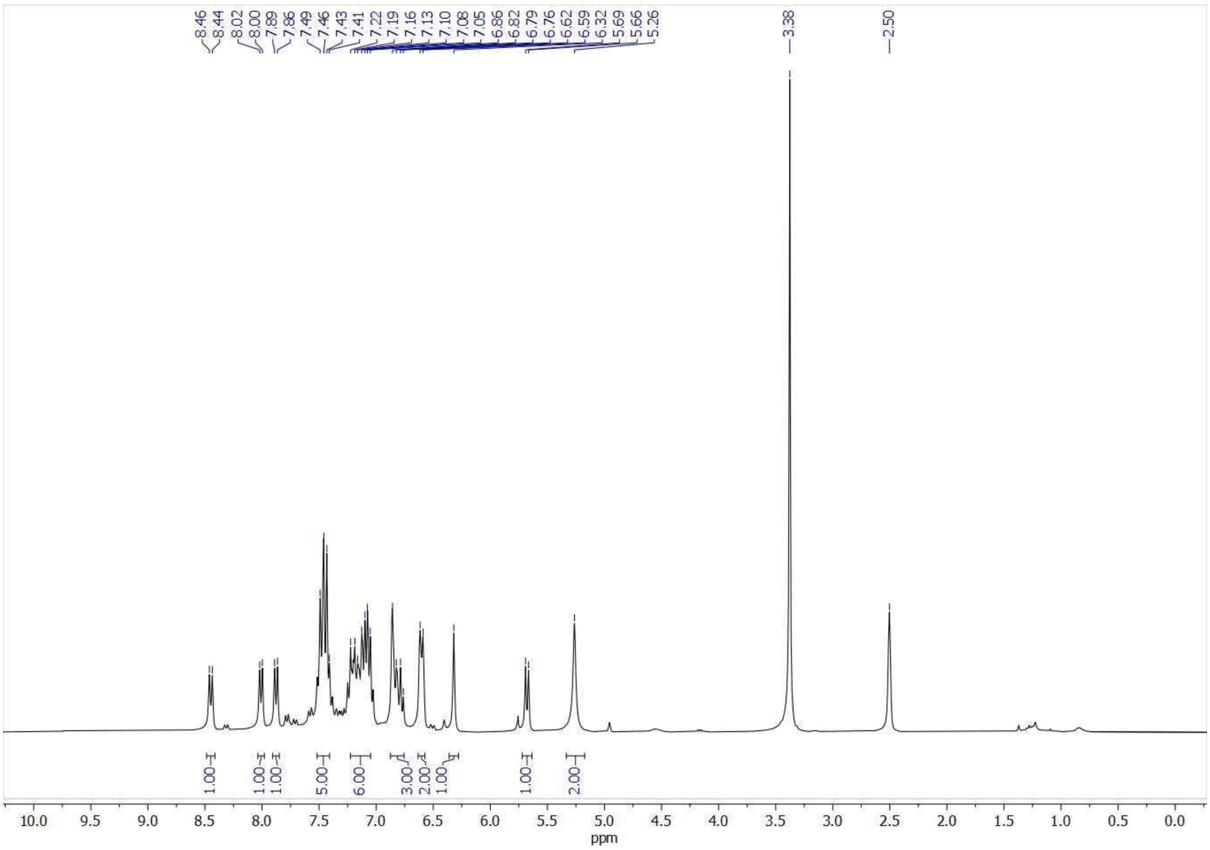

Figure S16. ^{1}H NMR spectrum of compound **4a** in DMSO-d_6.

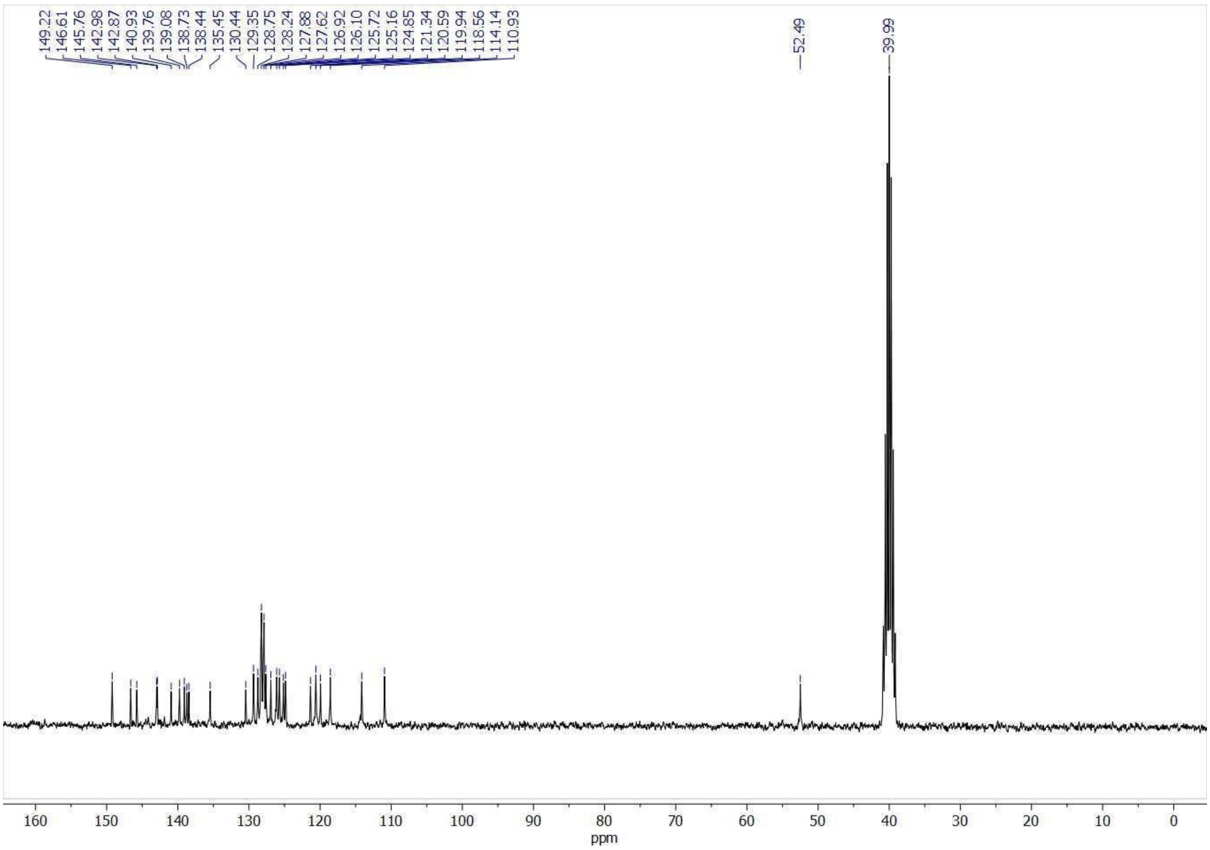

Figure S17. ^{13}C NMR spectrum of compound **4a** in DMSO-d_6.

Receptor R.

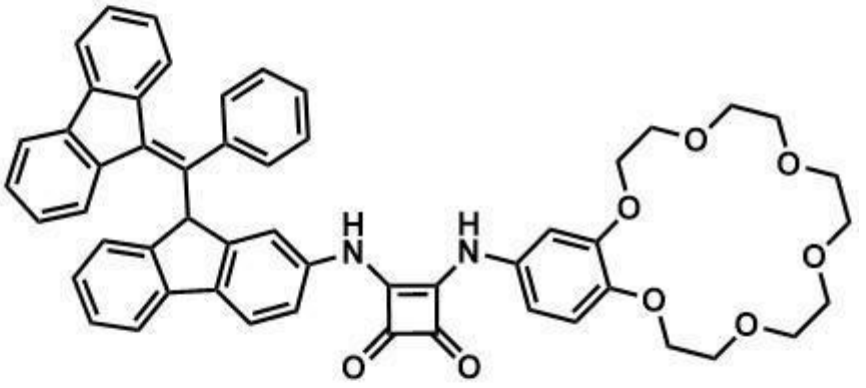

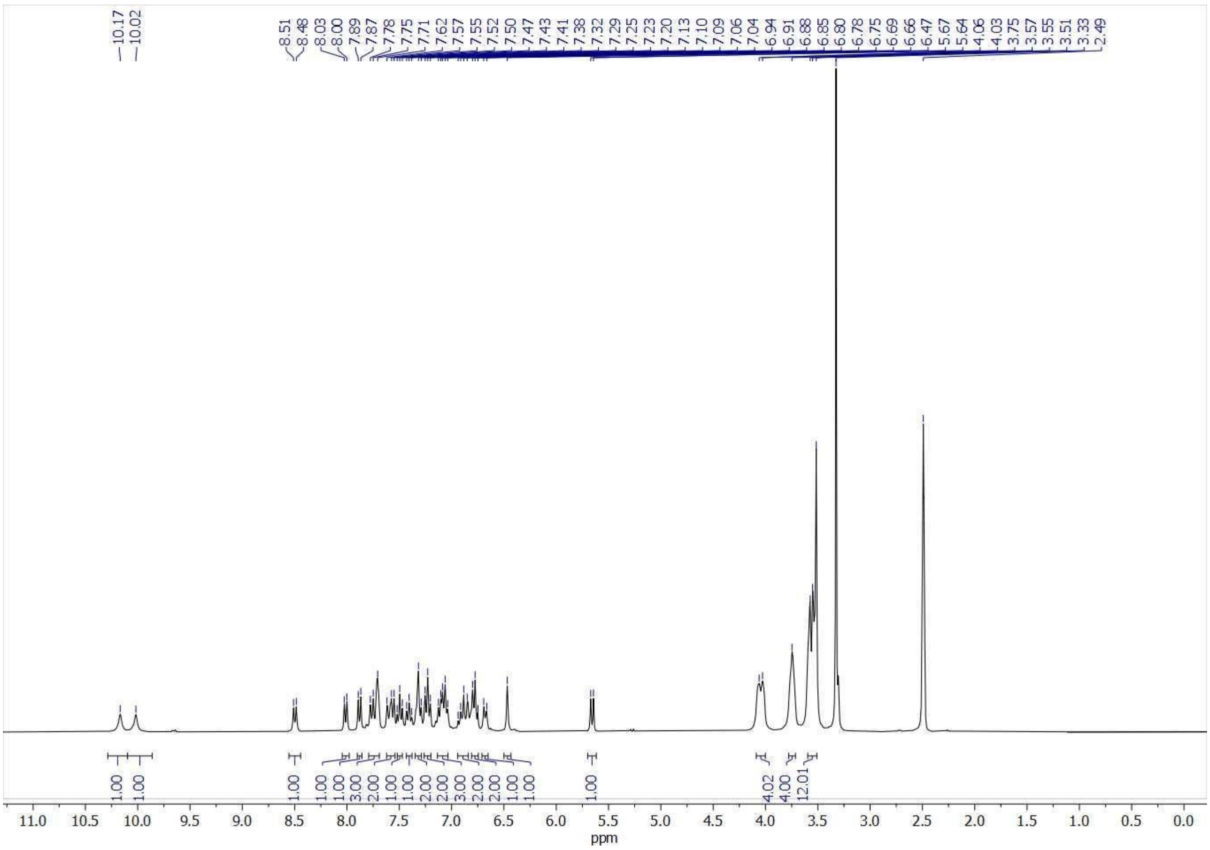

Figure S18. ^{1}H NMR spectrum of compound **R** in DMSO-d_6.

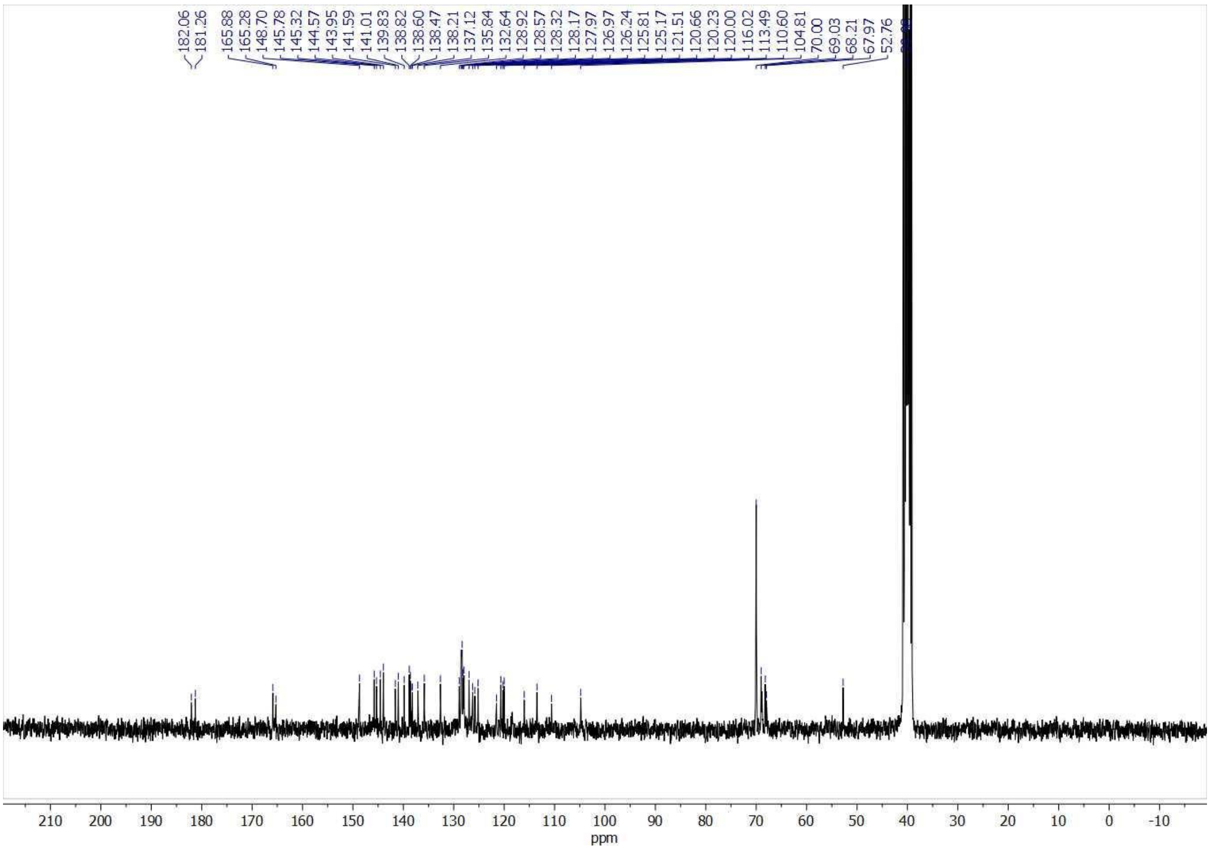

Figure S19. ^{13}C NMR spectrum of compound **R** in DMSO-d_6.

Radical R$^{\cdot}$.

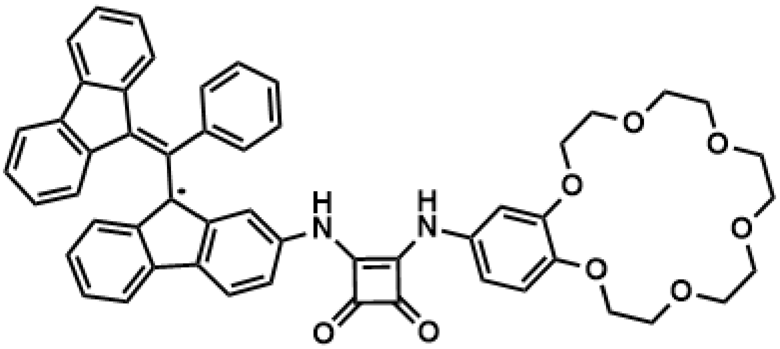

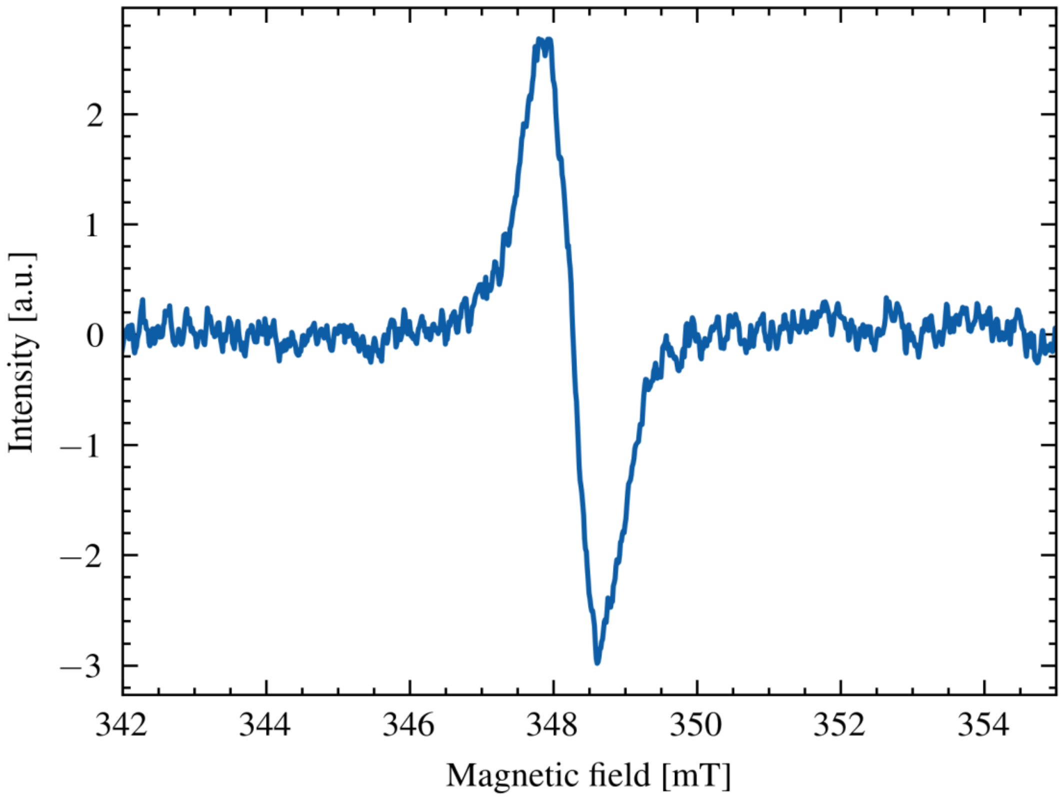

Figure S20. EPR spectrum of compound **R$^{\cdot}$** in DMSO.

BDPA-PMMA.

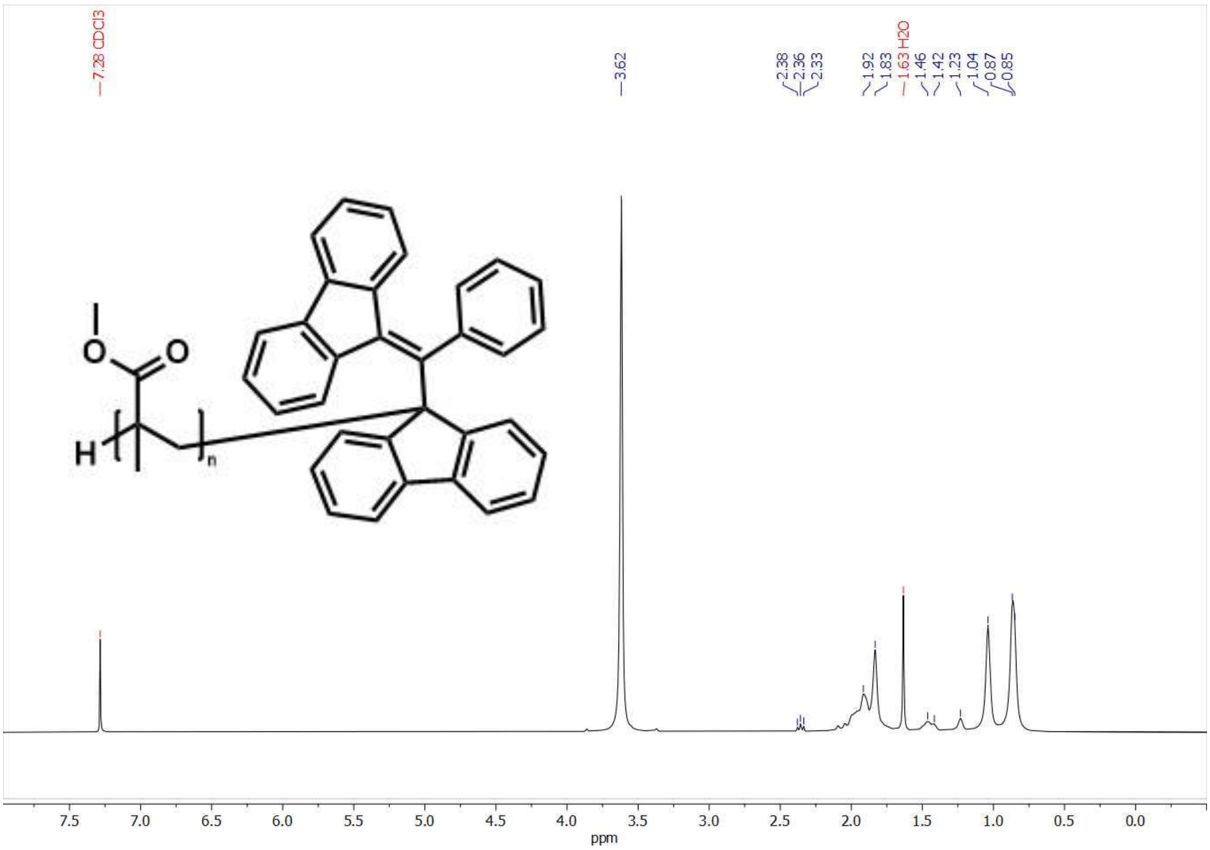

Figure S21. H[1] NMR spectrum of **BDPA-PMMA** in $CDCl_3$.

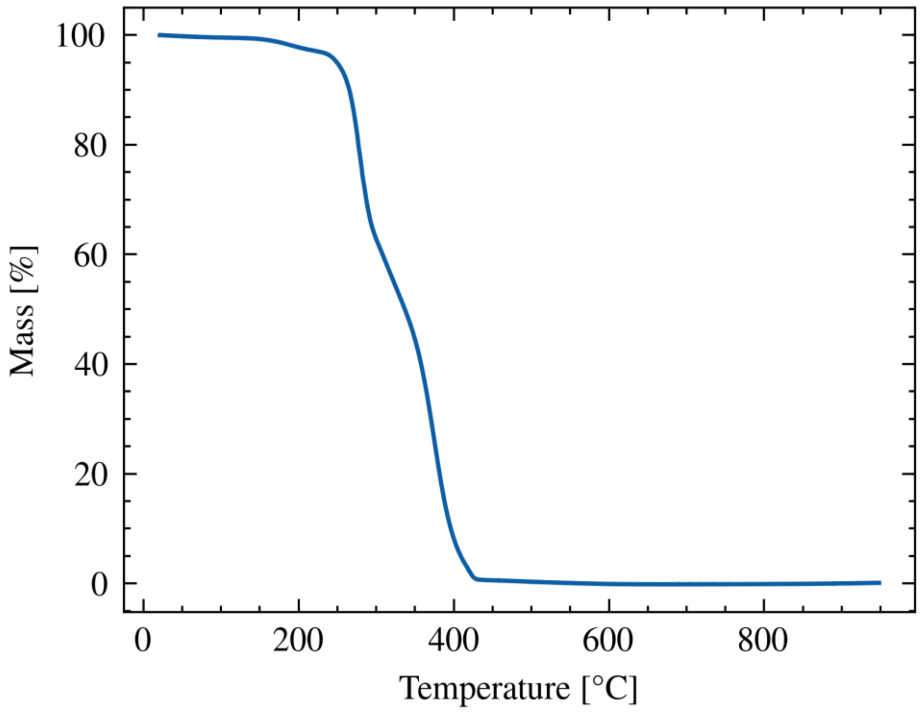

Figure S22. TGA curve recorded for **BDPA-PMMA**.

R-PMMA.

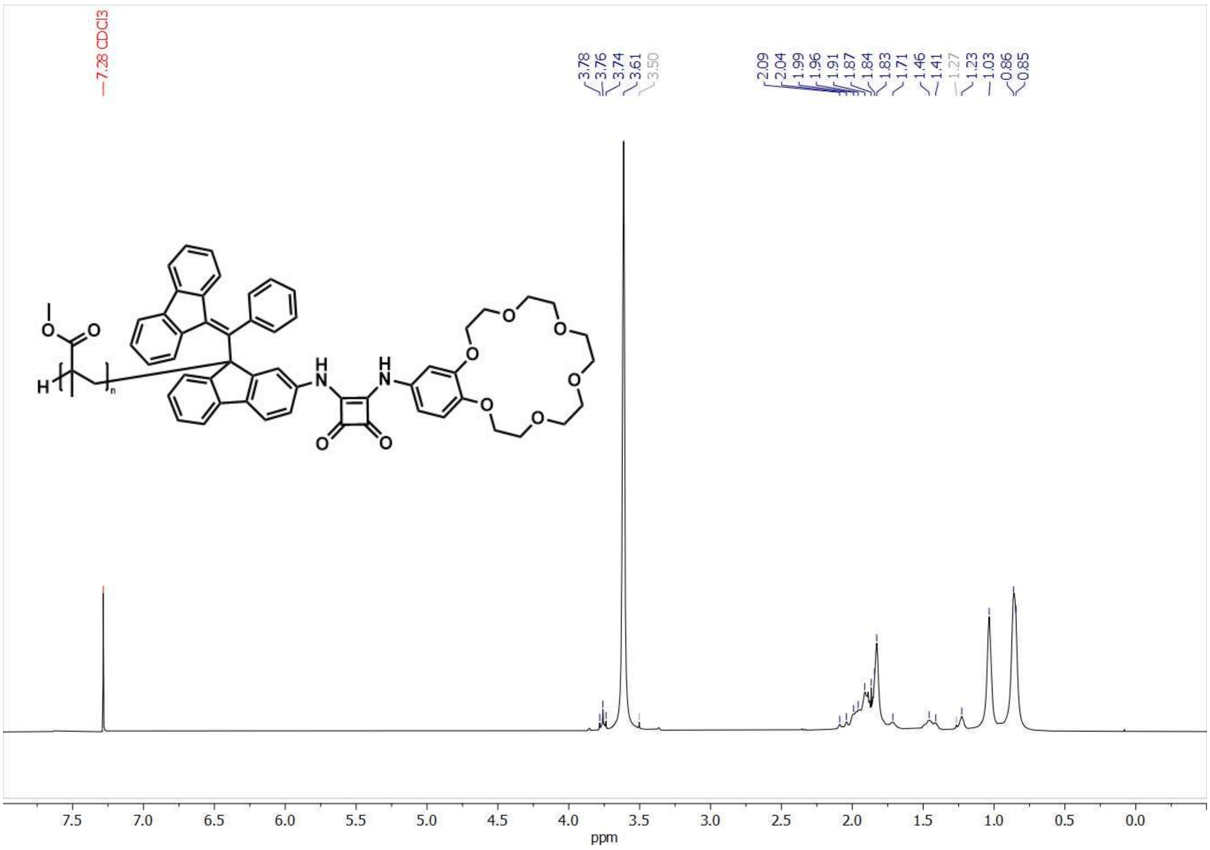

Figure S23. H^1 NMR spectrum of **R-PMMA** in $CDCl_3$.

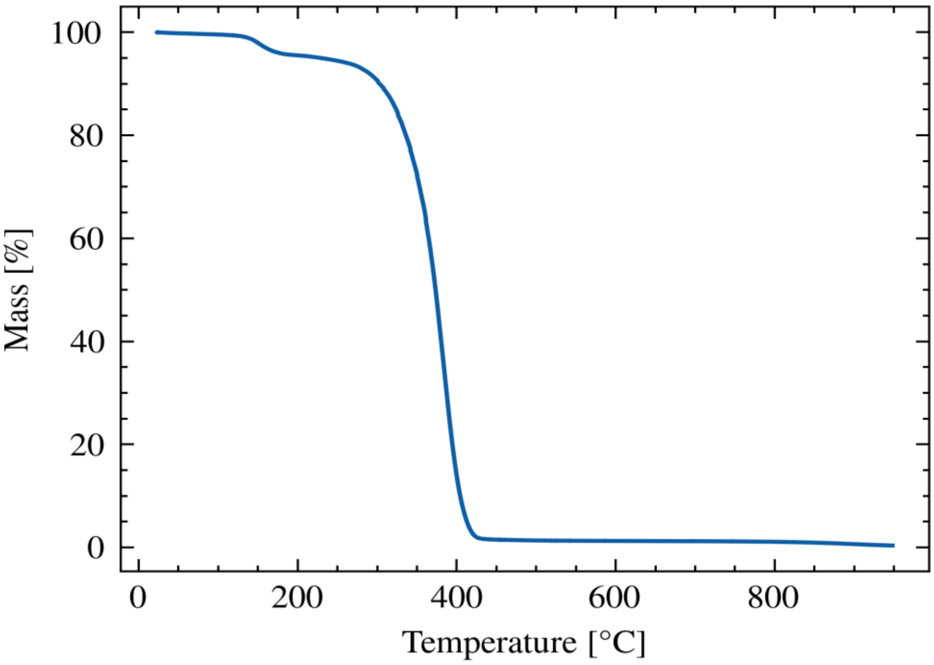

Figure S24. TGA curve recorded for **R-PMMA**.

3. UV-Vis titration experiments

UV-Vis experiment general procedure. UV-Vis titration experiments were performed on a Thermo Spectronic Unicam UV 500 spectrophotometer in 1% H_2O in MeCN solution at 298K. To a 10 mm cuvette 2.5 mL of freshly prepared 2×10^{-5} M solution of the receptor **R** was added and in the case of binding studies for salts, 1 equivalent of cation (KPF_6 or $NaClO_4$). Small aliquots of ~1.0×10^{-3} M TBAX solution containing receptor **R** at the same concentration as in the cuvette were added and a spectrum was acquired after each addition. The resulting titration data were analyzed using the BindFit (v0.5) package, available online at http://supramolecular.org. Alternatively, to a 10 mm cuvette was added 2.5 mL of PMMA solution in $CHCl_3$ (0.5 mg/ml **R-PMMA**, 1.5 mg/mL **BDPA-PMMA**), small aliquots of TBACl solution (~2.5×10^{-3} **R-PMMA**, ~5×10^{-3} **BDPA-PMMA**) containing their respective PMMA at the same concentration as in the cuvette were added and a spectrum was acquired after each addition.

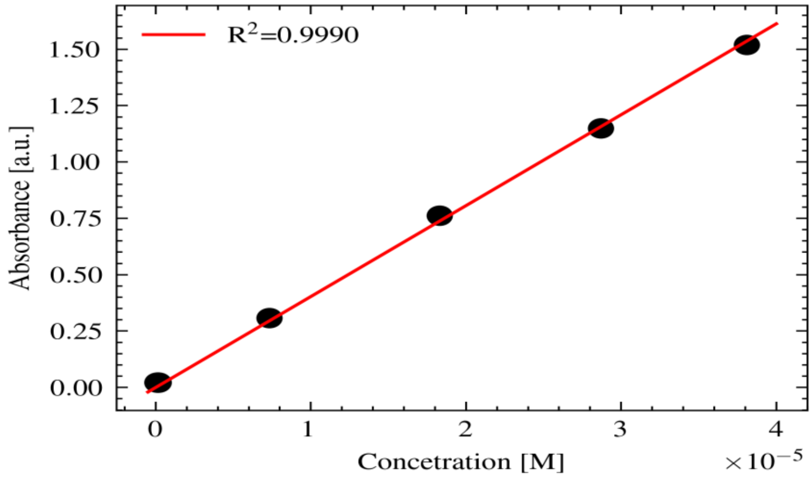

Figure S25. Dilution curve of receptor **R**.

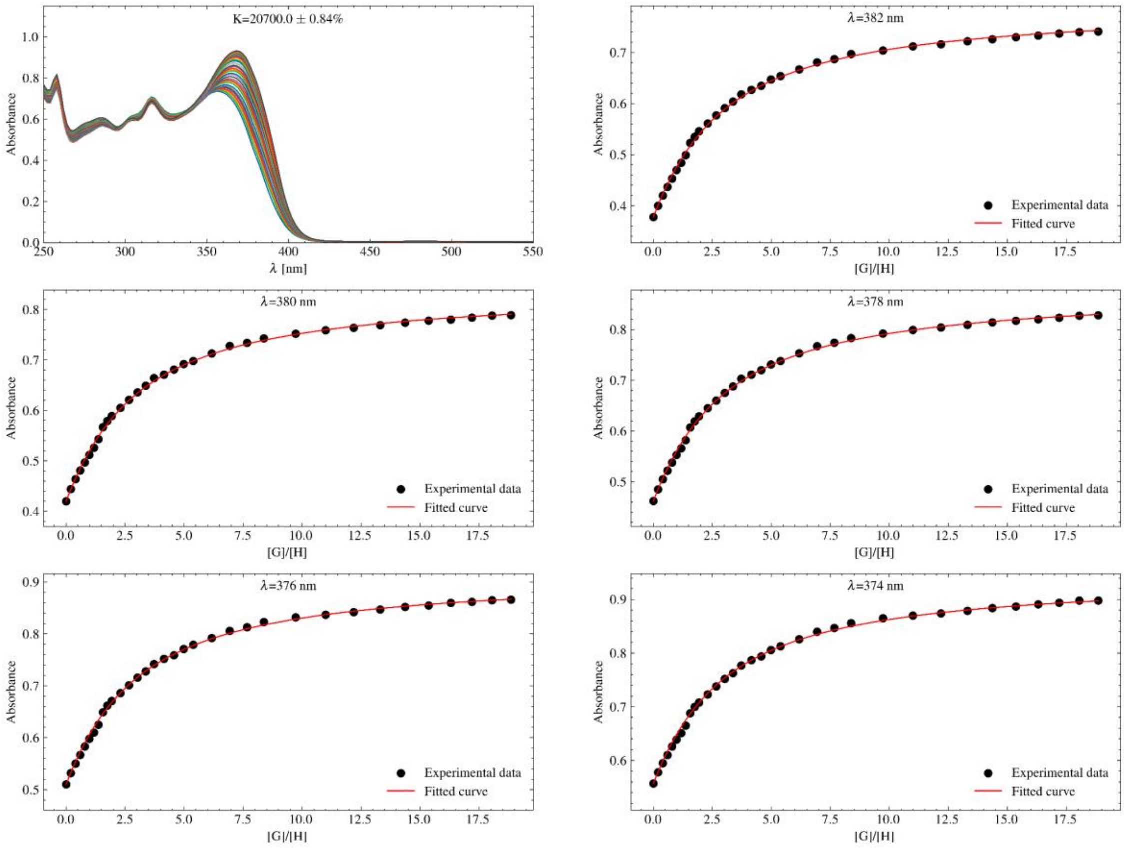

Figure S26. UV-Vis titration of receptor **R** with TBACl and selected binding isotherms.

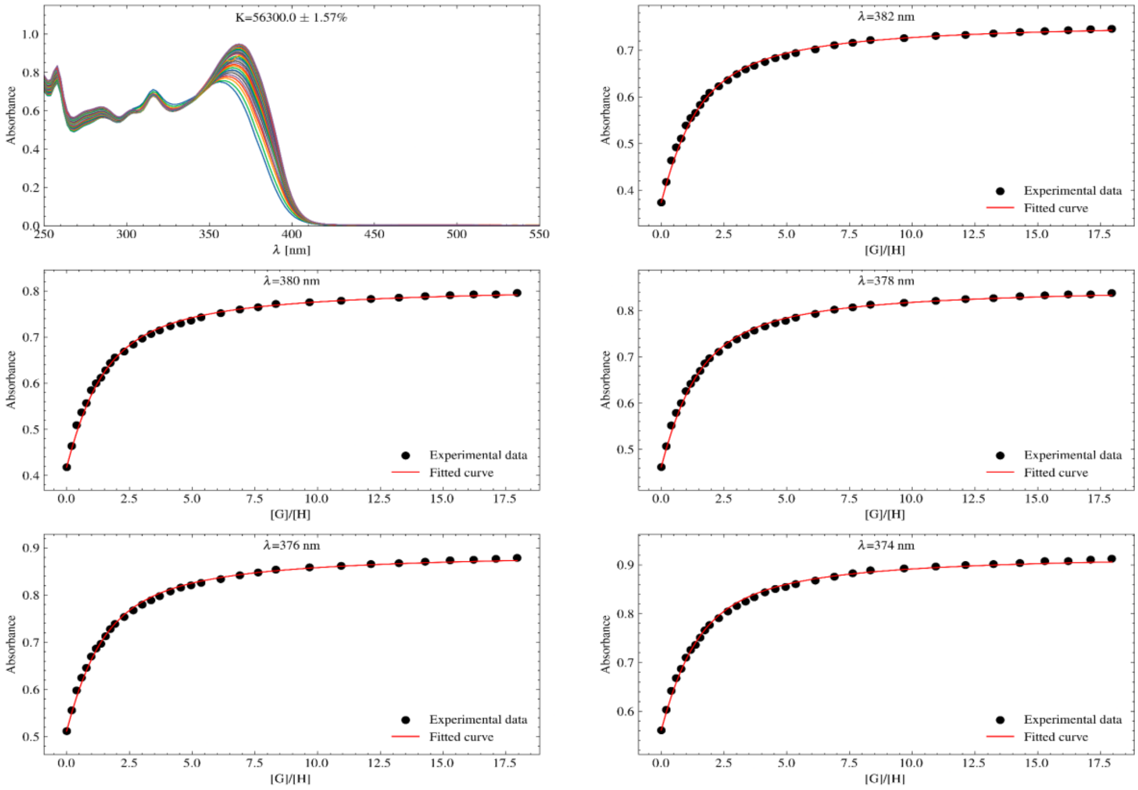

Figure S27. UV-Vis titration of receptor **R** with TBACl in the presence of 1 equivalent of $NaClO_4$ and selected binding isotherms.

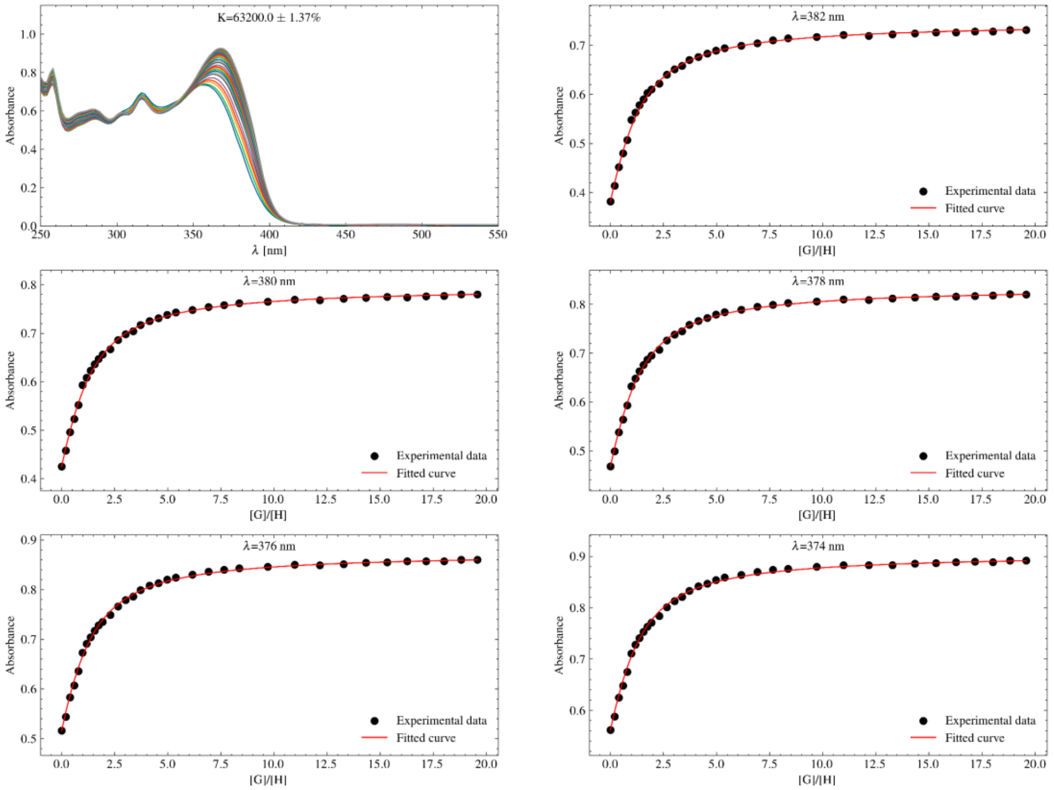

Figure S28. UV-Vis titration of receptor **R** with TBACl in the presence of 1 equivalent of KPF_6 and selected binding isotherms.

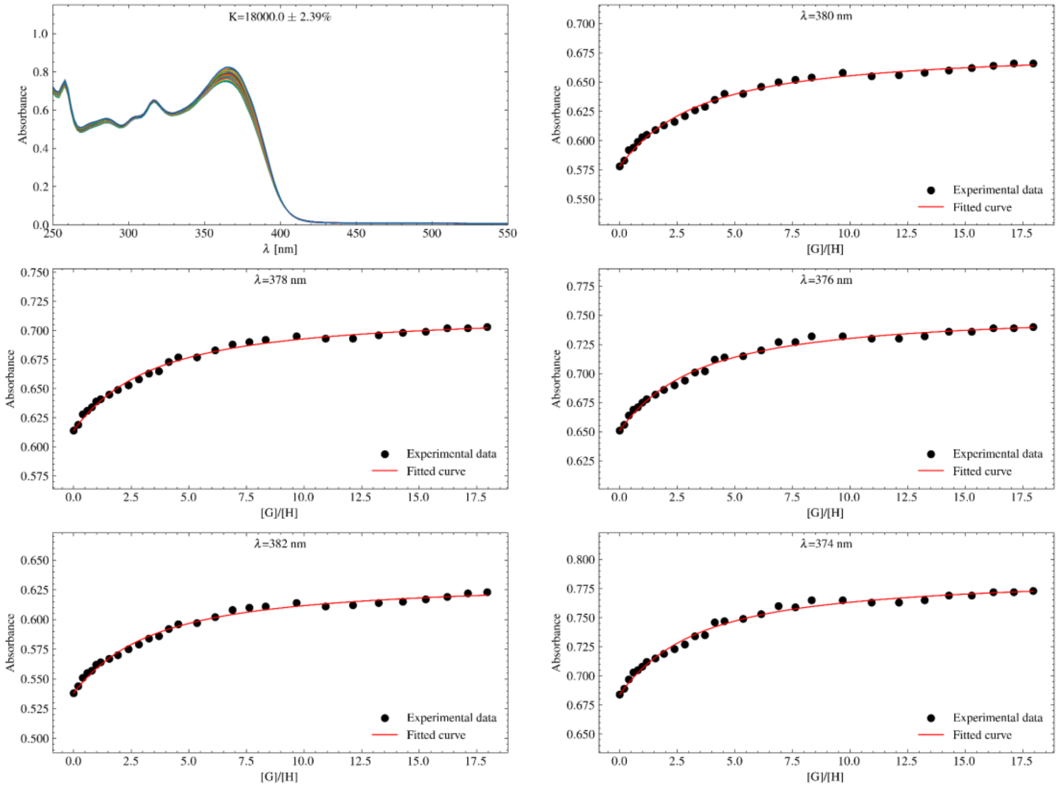

Figure S29. UV-Vis titration of receptor **R** with TBABr and selected binding isotherms.

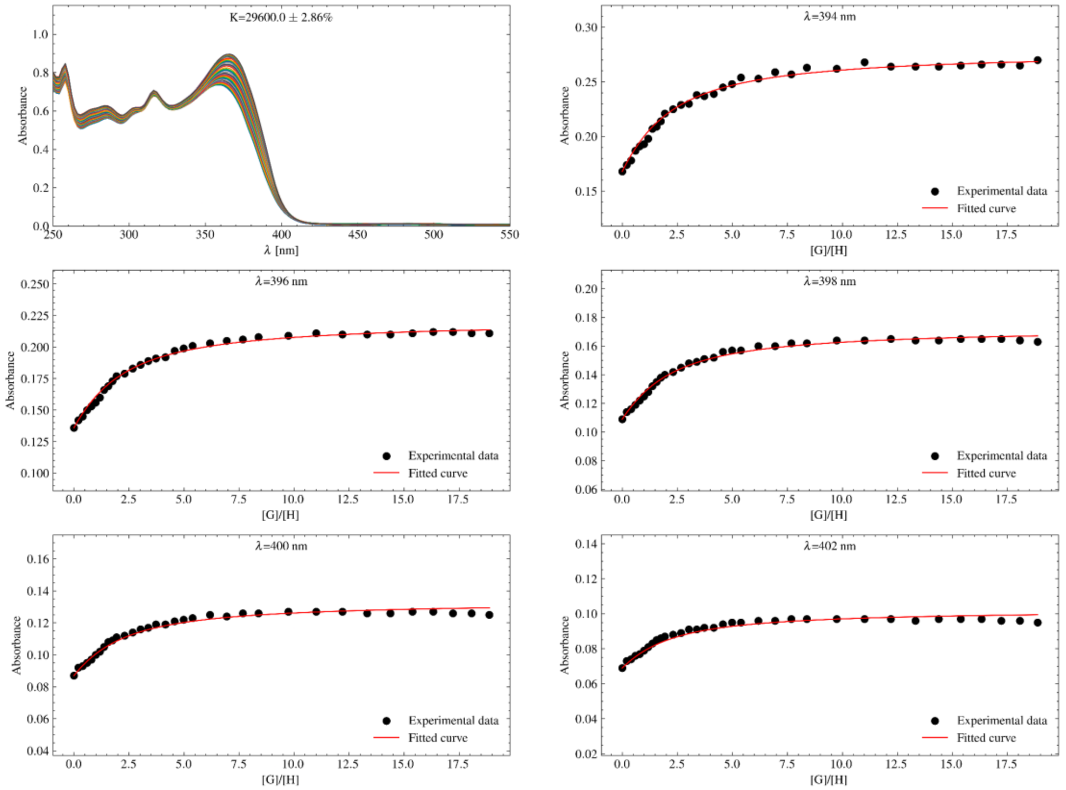

Figure S30. UV-Vis titration of receptor **R** with TBABr in the presence of 1 equivalent of KPF_6 and selected binding isotherms.

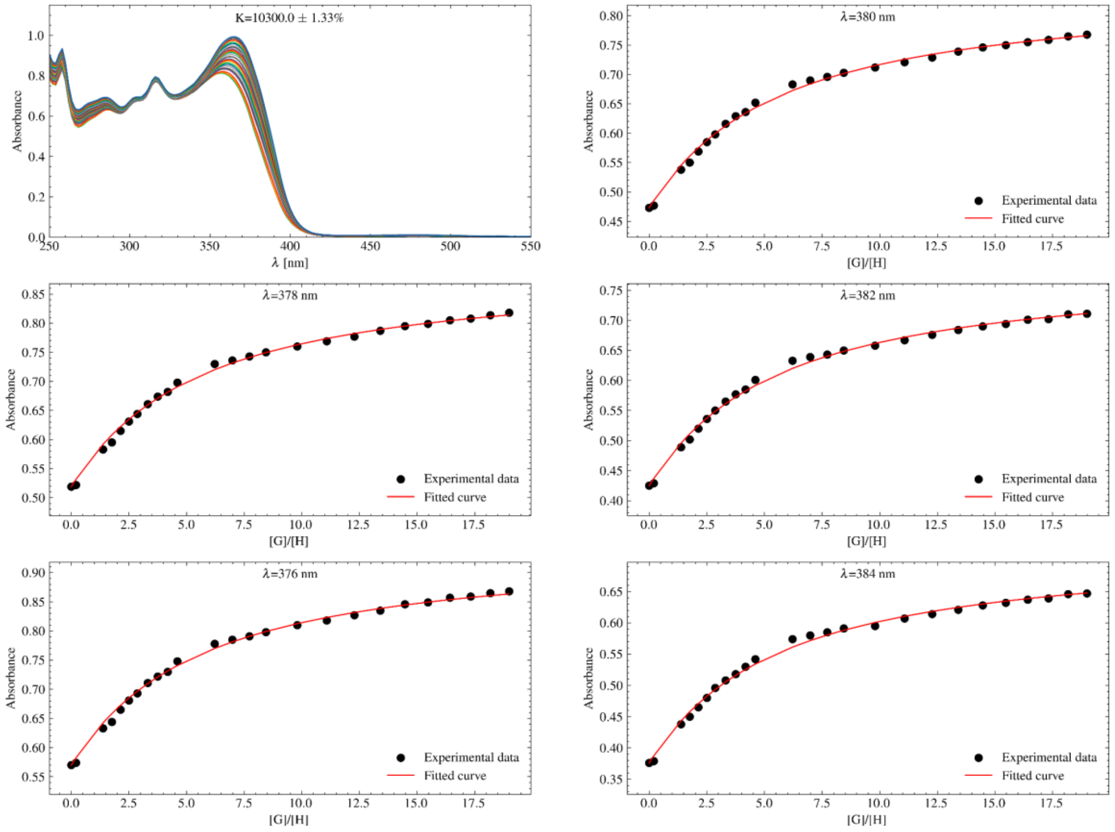

Figure S31. UV-Vis titration of receptor **R** with $TBANO_2$ and selected binding isotherms.

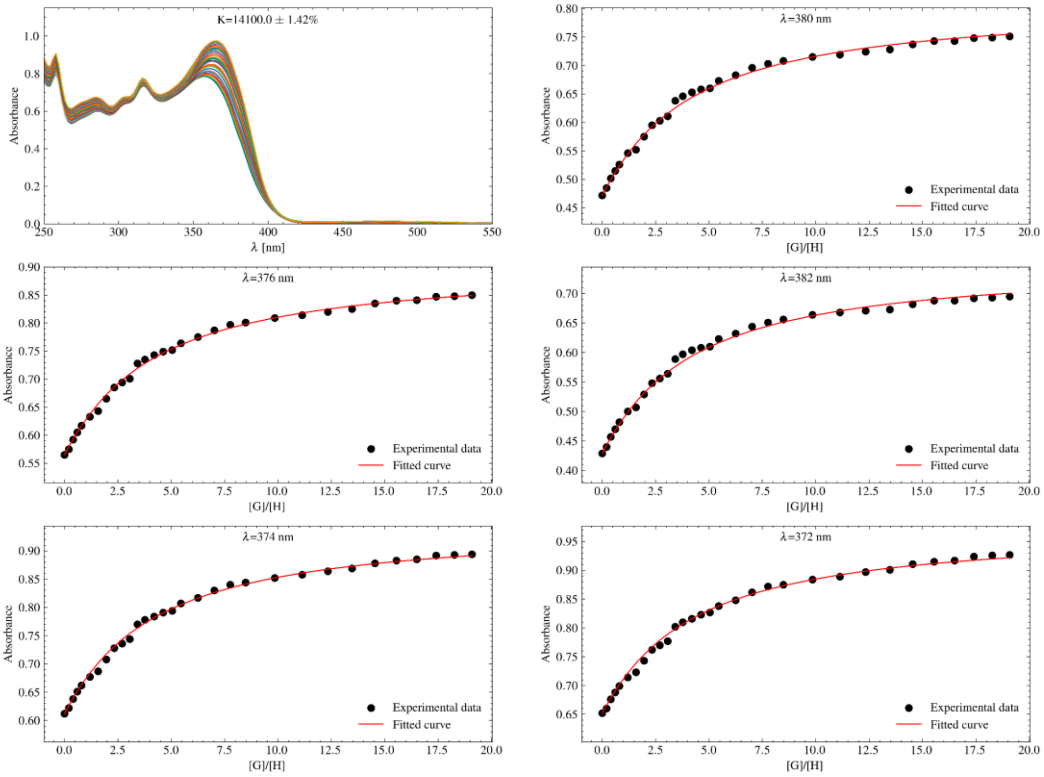

Figure S32. UV-Vis titration of receptor **R** with $TBANO_2$ in the presence of 1 equivalent of KPF_6 and selected binding isotherms.

4. NMR titration experiments

NMR experiment general procedure. ^{1}H NMR titration experiments were performed on a 300 MHz BrukerAvance spectrometer, at 298 K, in DMSO-d_6 solution. In each case, 0.5 mL of a 2.0 mM solution of receptor **R** was added to a 5 mm NMR tube. In the case of ion pair titration, the receptor was first pretreated with one equivalent of KPF_6. Then, small aliquots of solution of TBACl, containing receptor **R** at constant concentration, were added and a spectrum was acquired after each addition. The resulting titration data were analyzed using the BindFit (v0.5) package, available online at http://supramolecular.org.

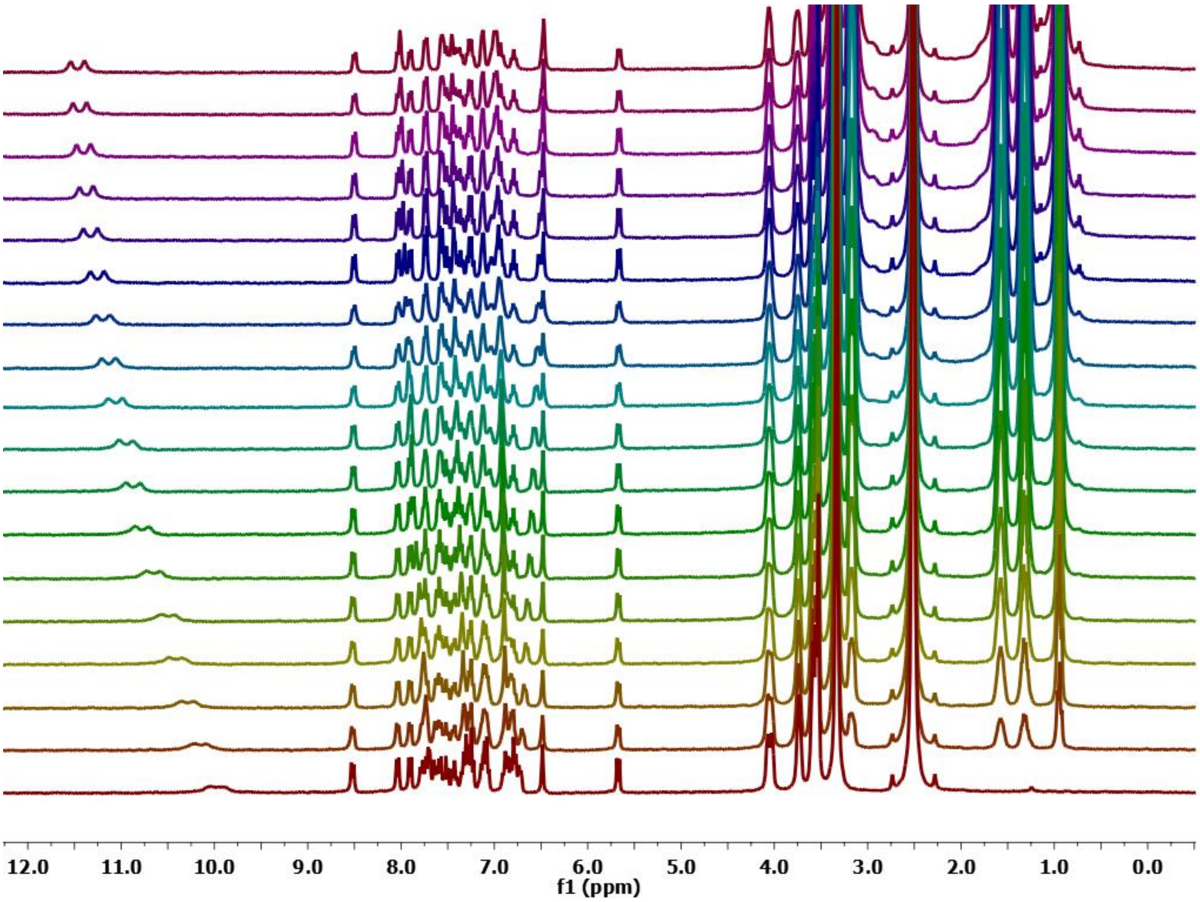

Figure S33. Typical ^{1}H NMR spectra recorded upon titration of **R** in DMSO-d_6 with TBACl.

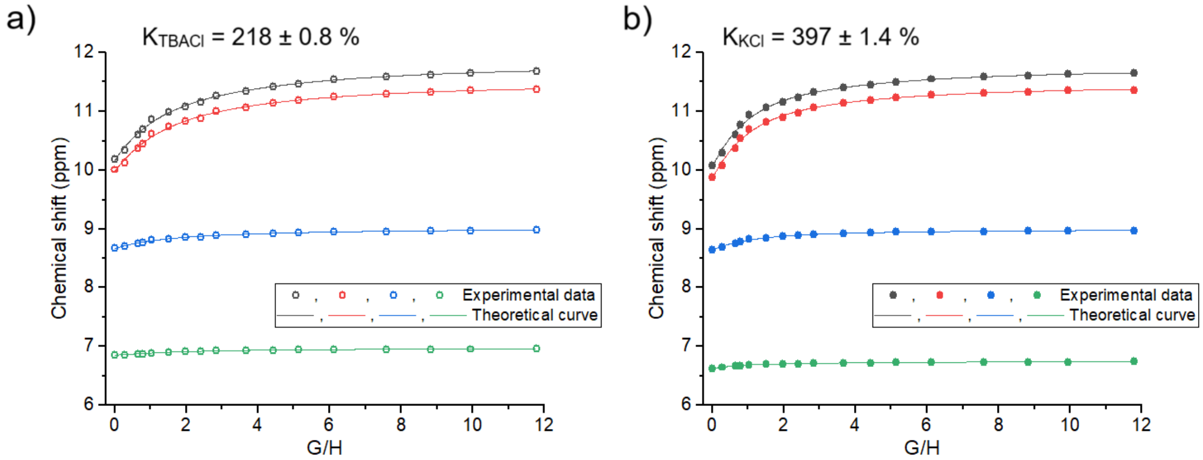

Figure S34. ^{1}H NMR titration binding isotherms of **R** in DMSO-d_6 a) upon the addition of increasing amounts of TBACl and b) of TBACl in the presence of 1 equiv. KPF_6.

5. Solid-liquid extraction experiments

Solid-liquid extraction experiment procedure. To 3 ml of **R-PMMA** or **BDPA-PMMA** solution (0.5 mg/ml) in $CHCl_3$ solid KCl (10 mg) was added and the solution was intensively stirred overnight. The solution was then filtrated through a syringe filter (0.22 μm), transferred to a 10 mm cuvette and a UV-Vis spectrum was recorded.

6. Liquid-liquid extraction experiments

Liquid-liquid extraction experiment procedure. To 3 ml of **R-PMMA** or **BDPA-PMMA** solution (0.5 mg/ml) in $CHCl_3$ 3 ml of aqueous KCl solution (1 M) was added and the mixture was intensively stirred overnight. The mixture was then separated, the organic phase was transferred to a 10 mm cuvette and a UV-Vis spectrum was recorded.

References

[S1] M. Plater, S. Kemp, E. Lattmann. "Heterocyclic free radicals. Part 1. 4,5-Diazafluorene derivatives of Koelsch's free radical: an EPR and metal-ion complexation study," in Journal of the Chemical Society, Perkin Transactions 1, no. 6, pp. 971–979, 2000.

[S2] Zaleskaya, M., et al. "Squaramide based ion pair receptors possessing ferrocene as a signaling unit," in Inorg. Chem. Front., vol. 7, pp. 972-983, 2020.
